# Selective and Reversible Solvent Uptake in Tetra‐4‐(4‐pyridyl)phenylmethane‐based Supramolecular Organic Frameworks

**DOI:** 10.1002/chem.202202977

**Published:** 2022-10-31

**Authors:** Danilo Marchetti, Francesca Portone, Francesco Mezzadri, Enrico Dalcanale, Mauro Gemmi, Alessandro Pedrini, Chiara Massera

**Affiliations:** ^1^ Department of Chemistry, Life Sciences and Environmental Sustainability INSTM UdR Parma University of Parma Parco Area delle Scienze 17/A 43124 Parma Italy; ^2^ Center for Materials Interfaces Electron Crystallography Istituto Italiano di Tecnologia Viale Rinaldo Piaggio 34 56025 Pontedera Italy

**Keywords:** host-guest systems, noncovalent interactions, stimuli-responsive crystals, supramolecular organic frameworks, 3D ED

## Abstract

The dynamic behavior of supramolecular organic frameworks (SOFs) based on the rigid tetra‐4‐(4‐pyridyl)phenylmethane (**TPPM)** organic tecton has been elucidated through 3D electron diffraction, X‐ray powder diffraction and differential scanning calorimetry (DSC) analysis. The SOF undergoes a reversible single‐crystal‐to‐single‐crystal transformation when exposed to vapours of selected organic solvents, moving from a closed structure with isolated small voids to an expanded structure with solvated channels along the *b* axis. The observed selectivity is dictated by the fitting of the guest in the expanded SOF, following the degree of packing coefficient. The effect of solvent uptake on **TPPM** solid‐state fluorescence was investigated, evidencing a significant variation in the emission profile only in the presence of chloroform.

## Graphical abstract

**TPPM‐based Supramolecular Organic Frameworks (SOFs**) undergo reversible single‐crystal‐to‐single‐crystal transformations when exposed to vapours of selected organic solvents and heat, respectively. The observed selectivity is dictated by the fitting of the guest in the expanded SOFs, following the degree of packing coefficient.

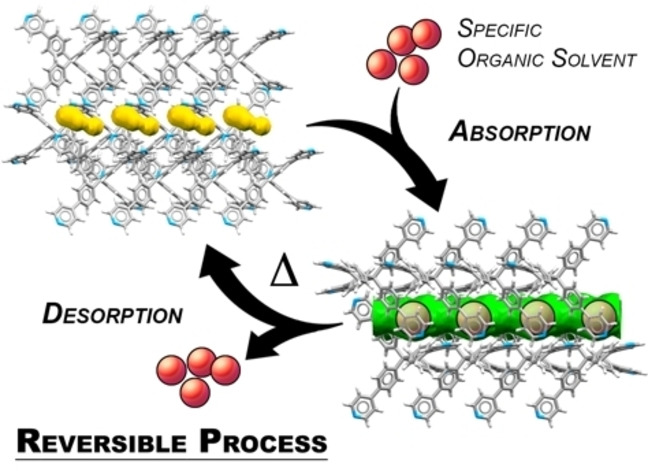

## Introduction

In recent years, supramolecular organic frameworks (SOFs) have emerged as an important class of functional porous materials alongside coordination polymers (CPs), metal–organic frameworks (MOFs) and covalent organic frameworks (COFs) for applications such as molecular sensing, gas storage and separation.[^1^, ^2^, ^3^, ^4^] Usually, SOFs are obtained through the self‐assembly of organic tectons via highly directional hydrogen bonds,[^5^, ^6^, ^7^, ^8^, ^9^, ^10^, ^11^, ^12^, ^13^, ^14^, ^15^, ^16^, ^17^, ^18^, ^19^] but examples of materials held together by van der Waals interactions, π⋅⋅⋅π stacking, halogen bonds and, quite recently, chalcogen bonds have also been reported.[^20^, ^21^, ^22^, ^23^, ^24^, ^25^, ^26^] Compared to other classes of reticular materials based on coordinative or covalent bonds, SOFs lack robustness and tend to loose porosity when guests molecules are removed.^[2]^ On the other side, they have the advantage of coupling flexibility and reversibility with relatively simple synthetic procedures under mild conditions.^[9]^ These considerations become especially important when targeting stimuli‐responsive crystalline materials, which respond to external solicitations such as electric, mechanical and thermal ones.[^27^, ^28^, ^29^, ^30^] The design of SOFs showing dynamic properties is a challenging task which involves both the synthesis of suitable building blocks, assembled through crystal engineering, and the mastering of molecular and supramolecular interactions in the solid state.[^3^, ^13^, ^22^, ^31^, ^32^, ^33^, ^34^]

**Scheme 1:**
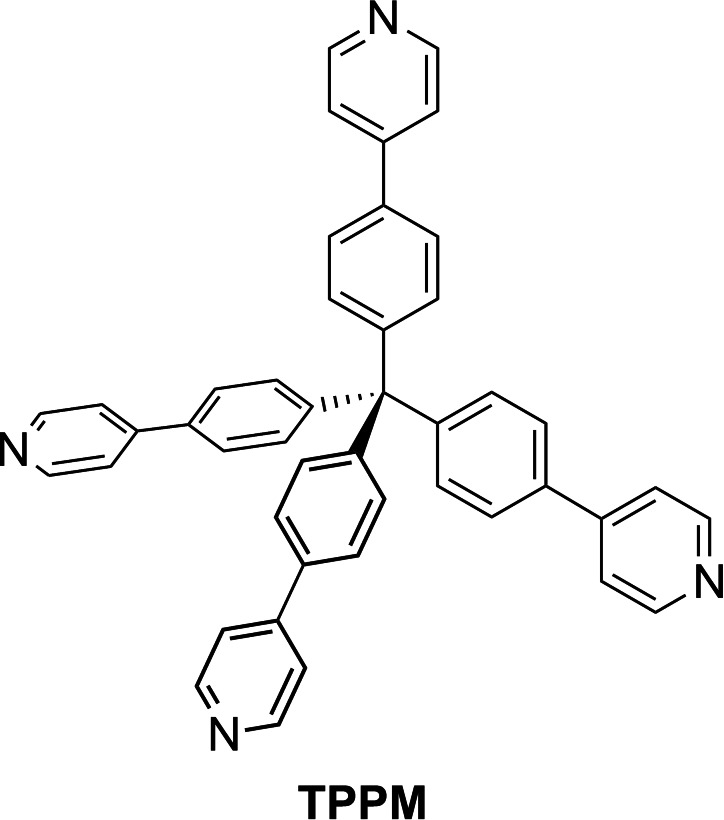
Molecular sketch of tetra‐4‐(4‐pyridyl)phenylmethane (**TPPM**).

Among the possible building blocks, tetraphenylmethane (**TPM**) derivatives, with their tetrahedral symmetry, represent effective tectons for the formation of 3D architectures.^[35]^ Exploiting their structural rigidity and synthetic versatility, these tectons have been successfully applied to the preparation of SOFs through the formation of highly oriented hydrogen bonding motifs based on 2‐pyridone,^[36]^ phenol,^[37]^ boronic acid,^[38]^ urethane^[39]^ and carboxylic acid^[40]^ functional groups. Instead, tetra‐4‐(4‐pyridyl)phenylmethane (**TPPM**, Scheme 1), the rigid aromatic tecton obtained by the decoration of **TPM** 4 positions with *p*‐substituted pyridyl rings, has been successfully employed for the synthesis of Cu‐based porous MOFs,[^41^, ^42^, ^43^, ^44^] but its potential as building block for SOFs is still unexplored. As shown by the electrostatic potential surface and by the Full Interaction Map^[45]^ (Figure S1), **TPPM** can act as hydrogen bond acceptor with its four nitrogen atoms, as weak hydrogen bond donor with the C−H moieties and can give rise to π⋅⋅⋅π and C−H⋅⋅⋅π interactions due to the presence of aromatic rings.

It is therefore expected that, according to Kitaigorodskii studies on organic crystals, the molecules assemble in the solid state to minimise free space.^[46]^ However, the relative weakness of the supramolecular interactions likely involved in stabilizing the crystal structure of **TPPM** enhances the probability of obtaining a flexible network^[47]^ susceptible of external, stimuli‐mediated structural changes.^[31]^

In this work, we present the dynamic behaviour of **TPPM** which, in its crystalline form, can reversibly switch from a non‐porous empty to a filled solvated phase and *vice‐versa*, when exposed to vapours of organic solvents and heat, respectively. Remarkably, this single‐crystal‐to‐single‐crystal transformation[^48^, ^49^, ^50^, ^51^] is selectively triggered only by a group of solvents, namely chloroform, dichloroethylene, trichloroethylene, benzene and toluene. In the specific case of chloroform uptake, a significant variation of the fluorescence emission profile was observed.

## Results and Discussion

**Figure 1:**
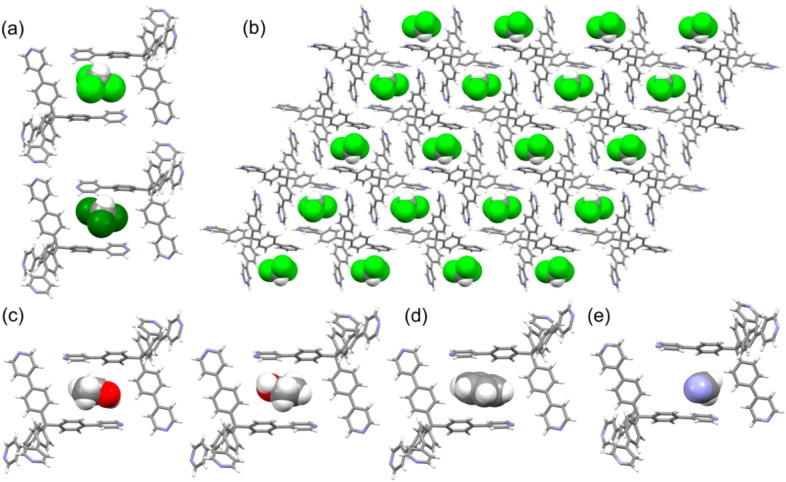
a) Detail of the two orientations of the chloroform guest obtained by rotation around the C−H bond. b) View along the *b*‐axis direction of the crystal lattice of **TPPM ⋅ CHCl_3_**. The solvent molecules filling the channels have been drawn in space filling mode. c) View of the two symmetry‐related orientations of ethanol in the crystal structure of **TPPM ⋅ EtOH**. d) View of benzene sandwiched between two **TPPM** ligands in **TPPM ⋅ C_6_H_6_**. e) View of acetonitrile inside the channels in **TPPM ⋅ 0.5CH_3_CN**.

To study the solid‐state behaviour of **TPPM**, crystals of the tecton were grown from the slow evaporation of a chloroform solution and analysed through X‐ray diffraction methods. **TPPM** crystallizes as a 1 : 1 CHCl_3_ solvate, **TPPM ⋅ CHCl_3_**, in the space group C2/*c*. The asymmetric unit consists of half a molecule of **TPPM** and half a molecule of the guest, disordered over two equivalent positions by a rotation around the C−H bond (Figure 1a). The **TPPM** units assemble to give a supramolecular three‐dimensional network held together by several C−H⋅⋅⋅N interactions. In particular, each **TPPM** forms symmetry‐related C12B−H12B⋅⋅⋅N1A, C3−H3A⋅⋅⋅N1B and C10−H10A⋅⋅⋅N1B contacts (Figure S2). This assembly contains channels parallel to the *b* axis direction filled by the disordered solvent molecules (Figure 1b). Inside the pores, chloroform forms two C−H⋅⋅⋅C_aromatic_ interaction involving the atoms C5A and C8A of **TPPM** (Figure S3).

Interestingly, the same supramolecular network also forms when **TPPM** is crystallized from different solvents, as reported in the literature for DMF^[41]^ (CSD^[52]^ refcode IVETEJ) and DMSO^[42]^ (CSD refcode: ZISXAC). In both isomorphous crystals, the solvent molecules occupy the channels in a 1 : 1 ratio. This prompted us to further study the robustness and the reproducibility of the SOF, by soaking single crystals of the chloroform solvate (for 2 h at room temperature) in different media such as ethanol, benzene and acetonitrile, potentially capable of forming hydrogen bonds or π⋅⋅⋅π stacking interactions with **TPPM**. In all three cases, the crystals retained their transparency and habit; X‐ray diffraction analysis confirmed the substitution of chloroform by the bulk solvents, and the formation of the three solvates **TPPM ⋅ EtOH**, **TPPM ⋅ C_6_H_6_** and **TPPM ⋅ 0.5CH_3_CN**. All solvates crystallize in the space group C2/*c* with comparable unit cell dimensions; **TPPM ⋅ EtOH** and **TPPM ⋅ C_6_H_6_** possess the same supramolecular network described above for **TPPM ⋅ CHCl_3_**, **TPPM ⋅ DMF** and **TPPM ⋅ DMSO** (Figure S4 and Table S1). The asymmetric unit of **TPPM ⋅ EtOH** consists of half a molecule of **TPPM** and half a molecule of the guest, which, due to symmetry, occupies the channels formed by the ligand with two different, symmetry‐related orientations (see Figure 1c). Analogously, the asymmetric unit of **TPPM ⋅ C_6_H_6_** consists of half a molecule of **TPPM** and half a molecule of the guest. Figure 1d shows benzene sandwiched between two symmetry‐related **TPPM** ligands, highlighting the shape complementarity of the guest with the channels formed by the host. In the case of **TPPM ⋅ 0.5CH_3_CN**, the asymmetric unit comprises half a molecule of **TPPM** and one fourth of a molecule of acetonitrile. Interestingly, **TPPM** is disordered over two positions, one of which is not compatible with the presence of the solvent; this means that in the crystal, both a solvate (Figure 1e) and an unsolvated (Figure S9b) phase are present. The N1S atom of acetonitrile forms a C−H⋅⋅⋅N interaction with a pyridine moiety (Figure S8). The framework of the solvated phase of **TPPM ⋅ 0.5CH_3_CN** still contains channels along the *b* axis direction, but it is consolidated by a different set of interactions, comprising also C−H⋅⋅⋅π⋅interactions (Figure S9a). The framework of the unsolvated phase, which is quite compact and contains unconnected voids summing up to 6 % of the unit cell volume, is also consolidated by C−H⋅⋅⋅N and C−H⋅⋅⋅π interactions (Figure S9b).

Crystals of **TPPM ⋅ CHCl_3_** were also exposed both to water vapours and soaked in distilled water for seven days to assess the stability of the solvate in humid and wet conditions. After these treatments, the X‐ray diffraction analysis of the crystals revealed the presence of the unaltered chloroform solvate.

The host‐guest interactions for the different solvates were compared by means of a Hirshfeld analysis, performed with CrystalExplorer17.^[53]^ The Hirshfeld surface of **TPPM** is quite similar for **TPPM ⋅ EtOH** and **TPPM ⋅ C_6_H_6_**, indicating that the presence of the guest does not influence much the supramolecular interactions consolidating the framework of the host (Figure S10). The Hirshfeld surface of **TPPM ⋅ CHCl_3_** shows an additional bright red spot relative to the C−H⋅⋅⋅C_aromatic_ interactions shown in Figure S3, while the red spots on the Hirshfeld surface of **TPPM ⋅ 0.5CH_3_CN** derive from the C−H⋅⋅⋅π interactions (Figure S9a) which are missing in the other solvates. For all the forms, the general trend is a predominance of dispersion forces and of H⋅⋅⋅H, C⋅⋅⋅H/H⋅⋅⋅C and N⋅⋅⋅H/H⋅⋅⋅N contacts (see fingerprint plots in Figure S11 and Table S2).

This peculiar behaviour prompted us to investigate the possibility to isolate an empty crystal structure of **TPPM** alone. To this purpose, a batch of single crystals of **TPPM ⋅ CHCl_3_** was heated at 100 °C for 2 h. Part of the batch was grinded and analysed through X‐ray powder diffraction; the comparison of the diffractograms of the chloroform solvate and the activated form clearly showed the formation of a new phase showing high micro‐crystallinity, probably due to the lack of disordered solvent inside the channels. The remaining single crystals show an increased mosaicity (Figure S12) which hampers the use of single crystal X‐ray diffraction and calls for 3D electron diffraction (3D ED),[^54^, ^55^, ^56^] which can be successfully employed with nanometric samples possessing small crystalline domains. Although **TPPM** crystals are sensitive to the electron beam, thanks to a special low dose set up in which the electron dose is reduced below 0.05 el s^−1^ Å^−2^ and the diffraction patterns are collected with a Timepix single electron detector,^[57]^ a full 3D ED experiment covering a 120° of reciprocal space can be performed without the amorphization of the crystals or a significant loss in resolution of the diffraction signal. The 3D ED data have been collected in precession electron diffraction mode (PEDT) and allowed an *ab‐initio* structure determination of **TPPM** using direct methods. Remarkably, the data quality was suitable for the dynamical structure refinement of the **TPPM** structure; dynamical refinement takes into account, in the modelling of diffracted intensity, the effect of dynamical scattering through a Bloch formalism.^[58]^ The structure is refined together with the thickness of the sample and both agreement factors and quality in the structure determination approach single crystal X‐ray precision.^[59]^ In the case of **TPPM**, dynamical refinement led to an improvement of the structural model and allowed to localize most of the hydrogen atoms (Figure S16). This is the first time that a SOF structure has been refined at a high precision taking into account dynamical scattering. The activated **TPPM**, which is indeed the empty phase, crystallizes in the monoclinic space group C2/*c* like the parent solvates. The biggest changes in the cell parameters involve the *a* axis, which goes from 27.9295(5) to 31.40(6) Å, the *β* angle, that likewise increases from 120.1390(10) to 133.1(1)° and the volume, which slightly decreases from 3628.2(1) Å^3^ to 3596(12) Å^3^ (see Table S1). In the lattice, each **TPPM** forms symmetry‐related C11B−H11B⋅⋅⋅Cg1 and C10A−H10 A⋅⋅⋅Cg2 contacts with four distinct adjacent molecules (Figure S17; Cg1 and Cg2 are the centroids of rings C8A−C12A/N1A and C8B−C12B/N1B, respectively). These interactions are lacking in the solvated forms and are responsible for the more compact structure of the framework. The same central reference molecule is also surrounded by another set of four ligands through weak, C−H⋅⋅⋅N interactions involving each of the pyridine rings, acting as H‐bond acceptors towards the C−H groups of the phenyl moieties. Also in this case, the pattern is rather different from that present in the solvated forms, where each **TPPM** unit behaves both as H‐bond donor and acceptor. The different interactions formed in the empty and solvated phase are also evidenced by the Hirshfeld surface analysis shown in Figure S10.

**Figure 2:**
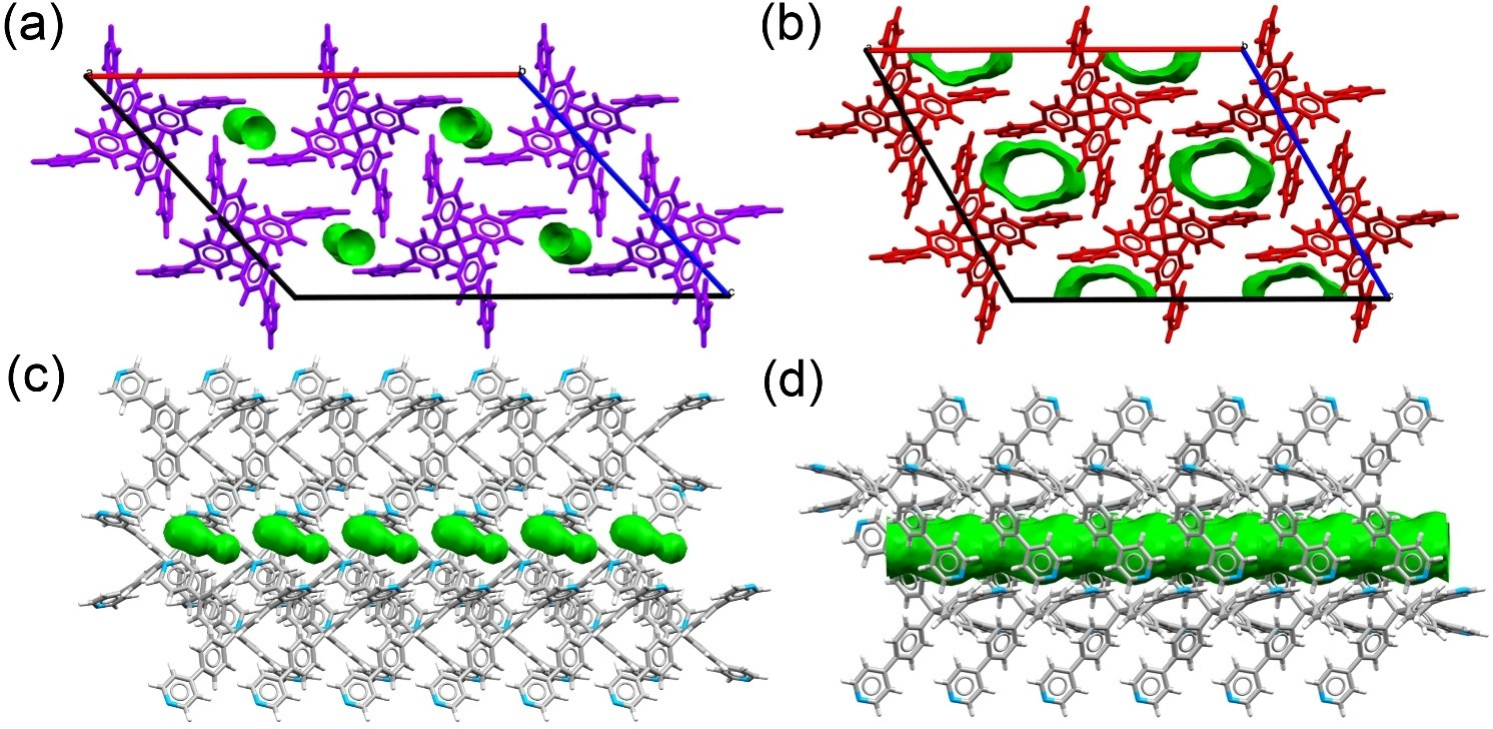
Comparison of the crystal lattice for the a) empty and b) solvated phases of **TPPM** and of their relative unconnected c) voids and d) channels. The solvent molecules of the solvated form have been removed for clarity.

The net result of this single‐crystal‐to‐single‐crystal phase transition is that of a compact framework in which the original channels become non‐connected small voids located among the **TPPM** units; these voids sum up to a volume of ca. 145 Å^3^ (4 % of the total unit cell volume) compared to the volume of ca. 610 Å^3^ (17 % of the total unit cell volume) occupied by the channels filled with solvent in the solvated form **TPPM ⋅ S** (Figure 2).

Interestingly, this framework is quite similar to the unsolvated form present in the **TPPM ⋅ CH_3_CN** crystals, which can be seen as a sort of intermediate phase in which, however, the unit cell is that of the solvated form.

**Figure 3:**
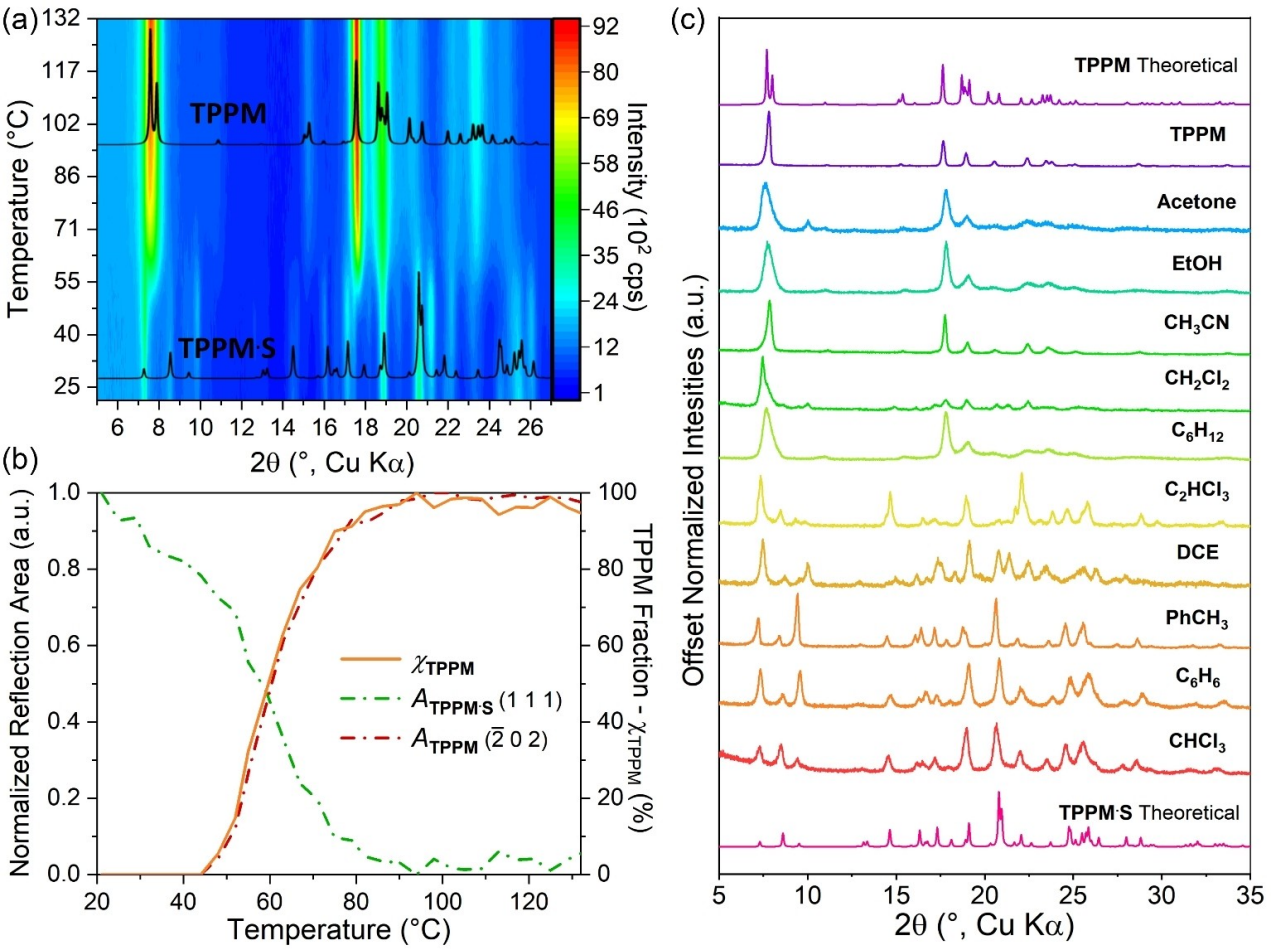
Temperature induced desolvation of **TPPM ⋅ S** analysed by temperature‐resolved *in situ* powder X‐ray diffraction for the chloroform solvate. a) 2D projection along the intensity axis of the powder diffractograms. The two peaks around 7° in the diffractogram of empty **TPPM** differ from the predicted pattern due to preferred orientation phenomena. b) Temperature‐resolved change in the peak area of (1 1 1) reflection of **TPPM ⋅ S** (A_TPPM‐S_) and ($\bar{2}$ 0 2) reflection of **TPPM** (A_TPPM_). *χ*_TPPM_ is the fraction of the empty **TPPM** phase. c) Powder X‐ray diffractograms after a 2‐minute exposure of the **TPPM** phase to vapours of different solvents.

The desolvation process was also monitored in detail through X‐ray powder diffraction analysis. To this purpose, the microcrystalline powder of **TPPM ⋅ CHCl_3_** was used as **TPPM ⋅ S** phase reference and measured by a temperature resolved *in situ* X‐ray powder diffraction analysis. The **TPPM ⋅ S** phase, during the heating treatment, undergoes a single‐crystal‐to‐single‐crystal phase transition leading to the empty **TPPM** phase. From the temperature‐resolved diffractograms it is evident that crystallinity is retained throughout the whole process (see Figures 3 and S18). The phase transition does not present a gradual change of lattice parameters, but only a reflection intensity change of the two involved species. LeBail refinement was performed for each collected diffractogram, leading to a temperature‐dependent correlation between powder profile parameters and the phase fraction (Figures 3 and S19).

**Figure 4:**
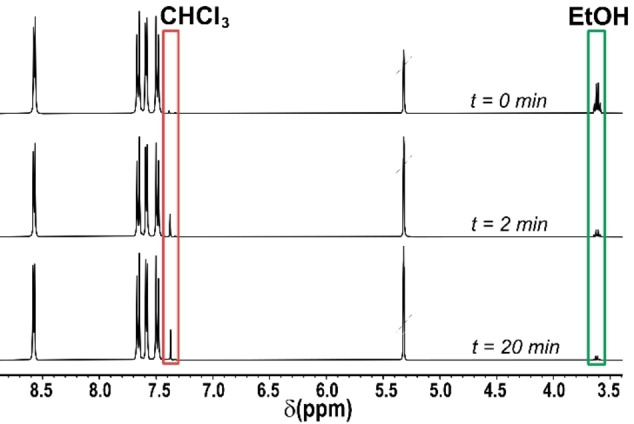
Solvent competition studies followed by ^1^H NMR spectroscopy. The **TPPM ⋅ EtOH** phase was exposed to CHCl_3_ vapours for 2 and 20 minutes. The solid was then dissolved in CD_2_Cl_2_ with few drops of methanol‐d_4_. ^1^H NMR spectra were collected and compared to the one of the initial solvate.

Interestingly, the dynamic behaviour shown by **TPPM** is reversible. Indeed, when the empty crystalline **TPPM** is exposed to the vapours of a series of organic solvents, it switches back to the solvated phase **TPPM ⋅ S**, as evidenced by PXRD analysis (Figures 3 and S20). The vapour uptake is fast (completed after ca. 2 minutes or less) and selective, as the phase transformation occurs with chloroform, dichloroethane, trichloroethylene, benzene, toluene but not with acetonitrile, ethanol, tetrachloroethylene or acetone. Cyclohexane also triggers the phase transformation, but its uptake time is much longer (ca. 20 minutes). Dichloromethane can be also partially absorbed by empty **TPPM** yielding, however, an unstable phase at room conditions. The affinity towards a group of solvent vapours shown by the SOF was also investigated by ^1^H NMR spectroscopy through competition experiments. Chloroform and ethanol were selected as representative of the two classes of vapours uptaken and not uptaken by the empty form. The exposure of the ethanol solvate for different intervals of time (2 and 20 minutes) caused the progressive substitution of ethanol inside the pores, as shown in the spectra reported in Figure 4 by the disappearance of its signals and the appearance of the chloroform ones. The reverse uptake did not occur when the crystalline **TPPM ⋅ CHCl_3_** solvate was exposed to EtOH vapours (Figure S35).

**Figure 5:**
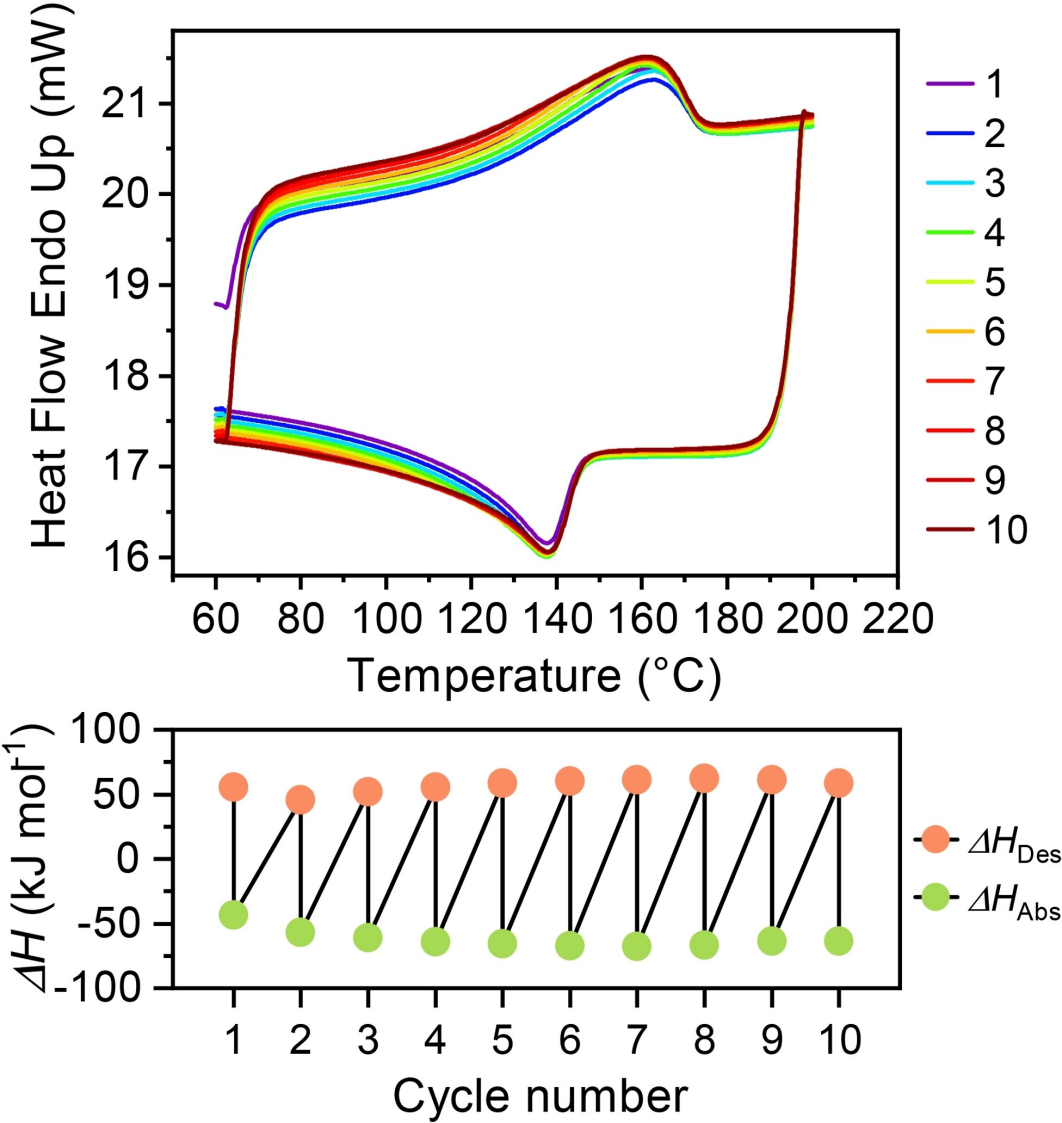
Absorption‐Desorption cycles performed through DSC analysis in a close system; the **TPPM**⋅**CHCl_3_** phase was used as reference. *Top*) Different thermograms collected for each cycle. *Bottom*) Enthalpy of desorption and absorption reported for each cycle.

The framework stability of the **TPPM ⋅ S** phases was investigated through DSC analysis. The characterizations were performed in an open system to minimize possible counterpressure effects. The collected thermograms present an endothermic phase transition, related to the release of guest molecules embedded in the **TPPM** framework (see Figures 5 and S21). It is worth noticing that the **TPPM ⋅ S** phases directly obtained by vapor absorption present higher desorption enthalpies (Δ*H*_Des_) than both **TPPM ⋅ EtOH** and **TPPM ⋅ 0.5CH_3_CN** (Figures S22 and S23) suggesting that thermodynamic stability can play a role in the selectivity of the absorption process. The release of solvent molecules during the phase transition was also confirmed by TGA (Figure S24) which, together with ^1^H NMR spectroscopy, was also used to determine the amount of absorbed solvent molecules, reported as solvate stoichiometry (Figures S25, S33 and S34). The dynamical behaviour of **TPPM** was studied performing the DSC analysis in a closed system, in which the solvent desorption is followed by the absorption process during the cooling path (Figure 5). This experiment was repeated several times to confirm the reversibility of the process; indeed, after each cycle the desorption (Δ*H*_Des_) and absorption (Δ*H*_Abs_) enthalpies did not show any significant change. This suggests that also the amount of absorbed and released guest after every repetition remains constant.

To further investigate the selectivity of the guests, their packing coefficient was calculated as the ratio between the guest volume (*V*_Guest_) and the volume of the guest‐binding site (*V*) present in the **TPPM ⋅ S** phase (see Section 5 in the Supporting Information). This parameter has been proposed by Rebek to predict the best binding accommodation of guests inside molecular capsules in solution, which is reached when the coefficient is ca. 55 %.^[60]^ Recently, this method has been shown to work nicely also for the absorption of gases inside the pores of supramolecular hosts.^[61]^ Indeed, it is remarkable that in our case, both CHCl_3_ and DCE show a packing coefficient of 55 and 56 %, respectively, and almost all the other guests that are absorbed by **TPPM** present packing coefficients in the range 60 %–70 %. On the contrary, all the solvents that are not uptaken or form unstable host‐guest complexes, show a low coefficient packing, typically in the range 35 %–44 %.

**Figure 6:**
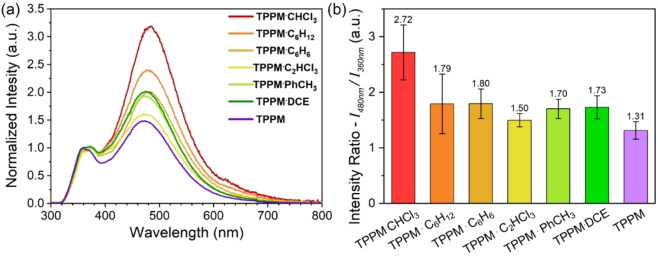
a) Comparison of the solid state emission spectra of the different **TPPM** solvates; b) diagram reporting the mean intensity ratio (*I*_480nm_/*I*_360nm_) values and their respective standard deviations.

The selective vapour uptake prompted us to investigate whether **TPPM** solid‐state spectroscopic properties are influenced by the nature of the absorbed solvent. With this aim, UV‐Vis absorption and emission spectra were recorded on powder samples of **TPPM** in thin layer form before and after their exposure to solvent vapours (Figures S37–S43). For all tested samples, the absorption profile (Figure S44) was found to be in good agreement with the one obtained for **TPPM** in DCM solution (Figure S36). On the contrary, in the emission spectra of the solid samples, the rising of a second, more intense band centred at 480 nm was observed (Figure 6a), in addition to the one at 360 nm characteristic of the solubilized **TPPM**. This behaviour is in line with the formation of excimers in tightly packed crystalline structures of aromatic molecules, as confirmed by the excitation profiles.^[62]^ Interestingly, the ratio between the emission intensity at the two maxima varies among all the series of tested solvates and differs from the **TPPM** empty form (Table S5). As depicted by the diagram reported in Figure 6b, the emission spectra were collected for three different samples and the significance levels for each mean value of intensity ratio was obtained by one‐way ANOVA calculation by Tukey Test. Only in the case of **TPPM ⋅ CHCl_3_** a significance level lower than the threshold value of 0.05 was recorded. These results make **TPPM** a potential candidate for the uptake and detection of chloroform vapours in air. As volatile organic compound (VOC), the release of chloroform in the atmosphere resulting from a wide range of industrial activities is of high interest and has a severe impact on both human and environmental health.^[63]^ In this respect, the stability of both **TPPM** and **TPPM ⋅ S** in presence of water is of particular relevance, as humidity is the main interferent in environmental monitoring of VOCs.

## Conclusion

In summary, we have elucidated the crystal structure of the solvated and empty forms of a crystalline SOF based on the rigid, tetrahedral organic tecton **TPPM**. This structural analysis, in which 3D ED has played an essential role, has paved the way for a deeper understanding of the dynamic behaviour of the material by means of X‐ray powder diffraction and DSC. This reversible single‐crystal‐to‐single‐crystal phase transition is remarkably triggered by a selective group of organic vapours; the origin of this selectivity has been also elucidated and lies in the difference of packing coefficient. In addition, we have shown that the solid‐state fluorescence of **TPPM** is affected by solvent uptake. The combined features of dynamic behaviour and selectivity can be exploited in the recognition of chloroform vapours through fluorescence measurements. We believe that this paper is another step towards a better comprehension of stimuli‐responsive materials held together by weak supramolecular interactions.

## Experimental Section

Synthesis, characterization and experimental details are provided as Supporting Information. This includes **TPPM** and SOFs preparation, detailed SCXRD, PXRD, 3D ED, DSC, TGA, NMR and spectroscopic studies and packing coefficient calculations.

Deposition Numbers 2194027 (for **TPPM ⋅ CHCl**_**3**_), 2194028 (for **TPPM ⋅ EtOH**), 2194029 (for **TPPM ⋅ C**_**6**_ **H**_**6**_), 2194030 (for **TPPM ⋅ 0.5CH**_**3**_ **CN**) and 2194031 (for **TPPM**) contain the supplementary crystallographic data for this paper. These data are provided free of charge by the joint Cambridge Crystallographic Data Centre and Fachinformationszentrum Karlsruhe Access Structures service.

## Conflict of interest

The authors declare no conflict of interest.

1

## Supporting information

As a service to our authors and readers, this journal provides supporting information supplied by the authors. Such materials are peer reviewed and may be re‐organized for online delivery, but are not copy‐edited or typeset. Technical support issues arising from supporting information (other than missing files) should be addressed to the authors.

Supporting Information

## Data Availability

The data that support the findings of this study are available from the corresponding author upon reasonable request.
