# Supplementary material for: Selective and Reversible Solvent Uptake in Tetra‐4‐(4‐pyridyl)phenylmethane‐based Supramolecular Organic Frameworks
Source: Chemistry. 2022 Oct 31;28(72):e202202977. doi: 10.1002/chem.202202977 (PMC10092063; doi:10.1002/chem.202202977)
Supplement: Supplementary file 1 — Supporting Information [file CHEM-28-0-s001.pdf]

Chemistry–A European Journal

Supporting Information

Selective and Reversible Solvent Uptake in Tetra-4-(4-pyridyl)phenylmethane-based Supramolecular Organic Frameworks

Danilo Marchetti, Francesca Portone, Francesco Mezzadri, Enrico Dalcanale, Mauro Gemmi, Alessandro Pedrini,* and Chiara Massera*

Table of contents

1. **Materials and Methods** S2

2. **Synthetic Procedures** S2
   - *2.1. **TPPM** Synthesis* S2
   - *2.2. Crystallization of **TPPM·$CHCl_3$*** S2
   - *2.3. Crystallization of **TPPM·C_6H_6*** S2
   - *2.4. Crystallization of **TPPM·EtOH*** S2
   - *2.5. Crystallisation of **TPP·ACN*** S3
   - *2.6. Solvent vapour absorption experiment* S3

3. **Solid state characterization** S4
   - *3.1. Single-crystal X-ray diffraction (SCXRD)* S4
   - *3.2. Hirshfeld surface analysis and fingerprint plots* S12
   - *3.3. 3D Electron diffraction (3D ED)* S14
   - *3.4. Powder X-ray diffraction (PXRD)* S17

4. **Thermal characterization** S21
   - *4.1. Differential Scanning Calorimetry (DSC)* S21
   - *4.2. Thermogravimetric Analysis (TGA)* S23

5. **Packing coefficients calculation** S25

6. **NMR Characterization** S26
   - *6.1. Solvent loading determination by 1H NMR spectroscopy performed on **TPPM·S** phases.* S27

7. **Spectroscopic Characterization** S32

8. **References** S39

1. Materials and Methods

All commercial reagents and solvents were used as received. Tetrakis(4-bromophenyl)methane, tetrakis(triphenilphenilphospine)palladium(0) end 4-pyridineboronic acid were purchased from TCI Europe. All instrumental details related to the adopted characterization techniques are reported in their respective sections.

2. Synthetic Procedures

*2.1. **TPPM** Synthesis*

The synthesis was performed following the procedure reported by Kitagawa et.al.[1] Tetrakis(4-bromophenyl)methane (400 mg, 0.63 mmol) and toluene (15 mL) were added to a 100 mL Schlenk tube, followed by an aqueous solution of sodium carbonate (667 mg, 6.29 mmol, in 5 mL of H_2O) and an ethanol suspension of 4-pyridineboronic acid (546 mg, 85% purity, 3.77 mmol, in 12 mL of ethanol). The mixture was purged with Ar (bubbling for 20 minutes) and tetrakis(triphenylphosphine)palladium(0) (87 mg, 0.07 mmol) was added. The reaction mixture was heated at 90 °C for 3 days under stirring. The mixture was cooled to room temperature and the precipitate was filtered. The off-white solid was washed with H_2O (3 × 3 mL) and dried. Recrystallization from a 9:1 CH_2Cl_2/MeOH mixture afforded TPPM as colorless crystals (240 mg, 61% yield).

^{1}H NMR (400 MHz, CD_2Cl_2) δ (ppm): 8.59 (dd; J_1 = 4.7 Hz, J_2 = 1.85 Hz, 8H), 7.66 (d, J = 8.6 Hz, 8H), 7.58 (dd, J1 = 4.7 Hz, J2 = 1.8 Hz, 8H), 7.49 (d, J = 8.5 Hz, 8H).

*2.2. Crystallization of **TPPM·CHCl$_3$***

TPPM (32 mg) was added to 20 mL of $CHCl_3$ in a 100 mL round bottom flask and left to reflux. The clear solution was cooled at room temperature and left to slowly evaporate, in order to obtain colorless needle crystals.

*2.3. Crystallization of **TPPM·C$_6$H$_6$***

Crystals of **TPPM·C$_6$H$_6$** were prepared starting from large crystals of **TPPM·CHCl$_3$**; they were added in a 5-mL scintillation vial with 3 mL of C_6H_6. The TPPM·$CHCl_3$ crystals were left to soak for 3 days yielding the **TPPM·C$_6$H$_6$** solvate.

*2.4. Crystallization of **TPPM·EtOH***

Crystals of **TPPM·EtOH** were prepared by soaking, following the same procedure described in section 2.3, using EtOH instead of C_6H_6.

*2.5. Crystallisation of **TPP·ACN***

Crystals of **TPPM·ACN** were prepared by soaking, following the same procedure described in section 2.3, using ACN instead of C_6H_6.

2.6. Solvent vapour absorption experiment

TPPM crystals were placed in an oven at 100°C to completely remove any traces of the solvent absorbed molecules. The activated **TPPM** crystals were subsequently placed in a closed vessel, previously saturated with vapours of a specific solvent and left for 2 minutes.

3. Solid state characterization

3.1. Single-crystal X-ray diffraction (SCXRD)

The crystal structures of **TPPM·CHCl$_3$**, **TPPM·EtOH**, **TPPM·C$_6$H$_6$** and **TPPM·0.5CH$_3$CN** were determined by X-ray diffraction on single crystals. The structure of the empty form of **TPPM** was solved by 3D Electron Diffraction (see section 3.3). Crystal data and experimental details for data collection and structure refinement are reported in Table S1. Intensity data and cell parameters were recorded at 200(2) K [167(2) K for **TPPM·0.5CH$_3$CN**] on a Bruker D8 Venture PhotonII diffractometer (CuKα radiation λ = 1.54178 Å). The raw frame data were processed using SAINT and SADABS to yield the reflection data files.[2] The structures were solved by Direct Methods using the SHELXT program[3] and refined on F_o^2 by full-matrix least-squares procedures, using SHELXL-2018[4] in the WinGX suite v.2014.1.[5] All non-hydrogen atoms were refined with anisotropic atomic displacements, with the exception of some of the disordered solvents. The hydrogen atoms were included in the refinement at idealized geometry and refined "riding" on the corresponding parent atoms. The weighting schemes used in the last cycle of refinement were $w = 1/ [\sigma^2 F_o^2 + (0.1661P)^2 + 5.9859P]$, $w = 1/ [\sigma^2 F_o^2 + (0.1710P)^2 + 4.3284P]$, $w = 1/ [\sigma^2 F_o^2 + (0.3487P)^2]$, and $w = 1/ [\sigma^2 F_o^2 + (0.2669P)^2]$, where $P = (F_o^2 + 2F_c^2)/3$, for **TPPM·CHCl$_3$**, **TPPM·EtOH**, **TPPM·C$_6$H$_6$** and **TPPM·0.5CH$_3$CN**, respectively. The crystallographic data have been deposited with the Cambridge Crystallographic Data Centre as supplementary publication no. 2194027, 2194028, 2194029 and 2194030.

Table S1. Crystallographic data for **TPPM·$CHCl_3$**, **TPPM·EtOH**, **TPPM·C_6H_6**, **TPPM·0.5CH_3CN**, **TPPM·DMF**[6], **TPPM·DMSO**[1] and **TPPM**.

	**TPPM·$CHCl_3$**	**TPPM·EtOH**	**TPPM·C_6H_6**	**TPPM·0.5CH_3CN**	**TPPM·DMF**	**TPPM·DMSO**	**TPPM**
Formula	$C_{45}H_{32}N_4·CHCl_3$	$C_{45}H_{32}N_4·C_2H_6O$	$C_{45}H_{32}N_4·C_6H_6$	$C_{45}H_{32}N_4·0.5(C_2H_3N)$	$C_{45}H_{32}N_4·C_3H_7NO$	$C_{45}H_{32}N_4·C_2H_6OS$	$C_{45}H_{32}N_4$
Formula weight	748.11	674.81	706.85	649.27	701.84	706.87	628.8
Crystal system	Monoclinic	Monoclinic	Monoclinic	Monoclinic	Monoclinic	Monoclinic	Monoclinic
Space group	*C*2/c	*C*2/c	*C*2/c	*C*2/c	*C*2/c	*C*2/c	*C*2/c
a/Å	27.9292(5)	28.0868(10)	28.074(2)	28.194(3)	27.985(4)	27.918(3)	31.4066
b/Å	6.9946(1)	7.0429(2)	7.0379(4)	7.0654(7)	7.0302(9)	6.9508(6)	7.1129
c/Å	21.4760(4)	21.2863(8)	21.3400(10)	21.254(3)	21.501(3)	21.315(2)	22.063
α/°	90	90	90	90	90	90	90
β/°	120.139(1)	120.765(1)	120.782(4)	122.335(7)	121.0620(10)	119.427(4)	133.175
γ/°	90	90	90	90	90	90	90
V/Å^3	3628.2(1)	3618.1(2)	3622.4(4)	3577.2(7)	3623.55	3602.58	3594.34
Z	4	4	4	4	4	4	4
D_c/g cm^{-3}	1.370	1.239	1.296	1.206	1.286	1.303	1.162
F(000)	1552	1424	1488	1364	1480	1488	1320
μ/mm^{-1}	2.598	0.580	0.585	0.552	0.078	0.134	-
$\theta_{min,max}$/°	3.66, 79.57	3.66, 72.43	3.66, 72.50	3.71, 59.2	3.02, 25.00	2.19, 30.05	0.12, 1.2
Reflections collected/unique	42954 / 3913 [R(int) = 0.0380]	38183 / 3582 [R(int) = 0.0579]	39587 / 3592 [R(int) = 0.0693]	14926/2569 [R(int) = 0.0855]	16397 / 3169 [R(int) = 0.0497]	38910 / 5272 [R(int) = 0.0343]	31938
R [Fo>4σ(Fo)]^a, wR_2^{a}	0.0796, 0.2499	0.0706, 0.2362	0.0955, 0.3452	0.1089, 0.3152	0.0451, 0.1057	0.0604, 0.1530	0.1148, 0.1302

$^aR_1 = \Sigma\|F_o| - |F_c\|/\Sigma|F_o|$, $wR_2 = [\Sigma[w(F_o^2\ F_c^2)^2]/\Sigma[w(F_o^2)^2]]^{1/2}$

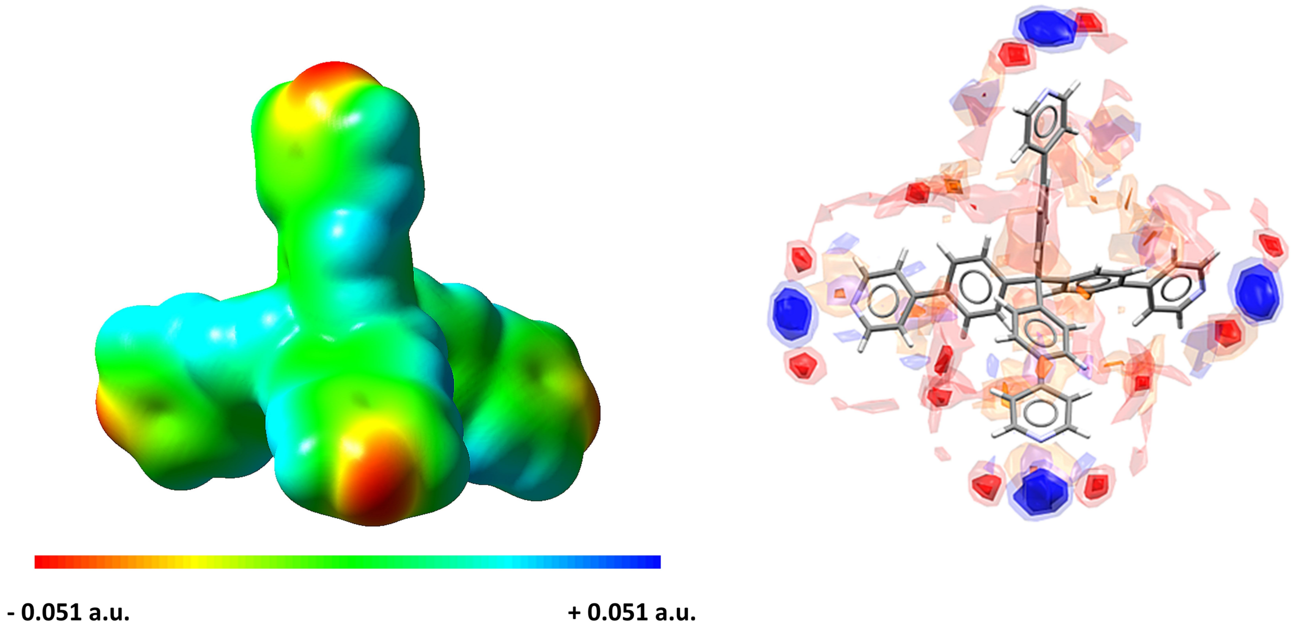

Figure S1. Electrostatic potential surface (left) and Full Interaction Map (right) for **TPPM**. The surface has been calculated at the B3LYP/6-311++G(d,p) level of theory using Gaussian09.[7] The colour code from red to blue indicate areas of rich and depleted electron density, respectively. In the Full Interaction Map, the different contour surfaces are used to indicate the likelihood of the molecule to form interactions with H-bond donors (in blue), acceptors (in red) and hydrophobic groups (in orange).

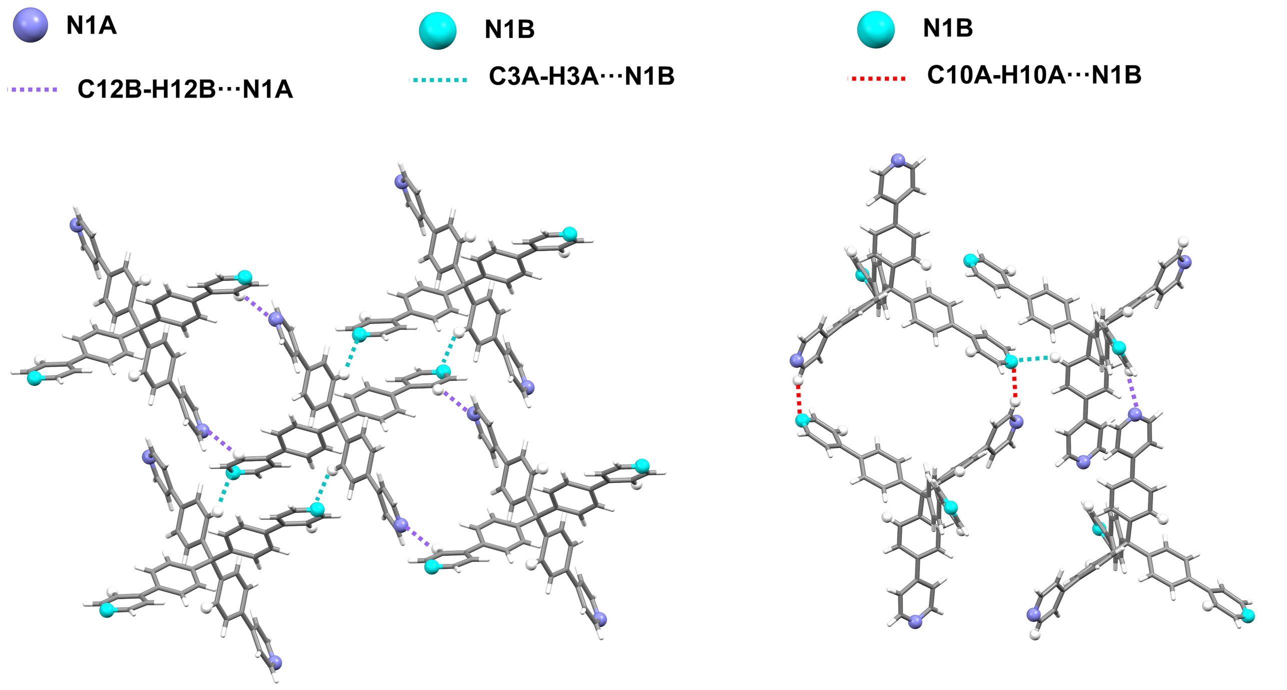

Figure S2. View of the relevant C-H···N interactions in **TPPM·CHCl$_3$**. The N and H atoms involved in the interactions have been drawn as spheres. Solvent molecules have been omitted for clarity. C12B··N1A and·C12B-H12B···N1A: 3.448(3) Å and 136.4(5)°; C3A···N1B and·C3A-H3A···N1B: 3.537(2) Å and 141.9(6)°; C10A···N1B and·C10A-H10A···N1B: 3.346(2) Å and 151.9(6)°.

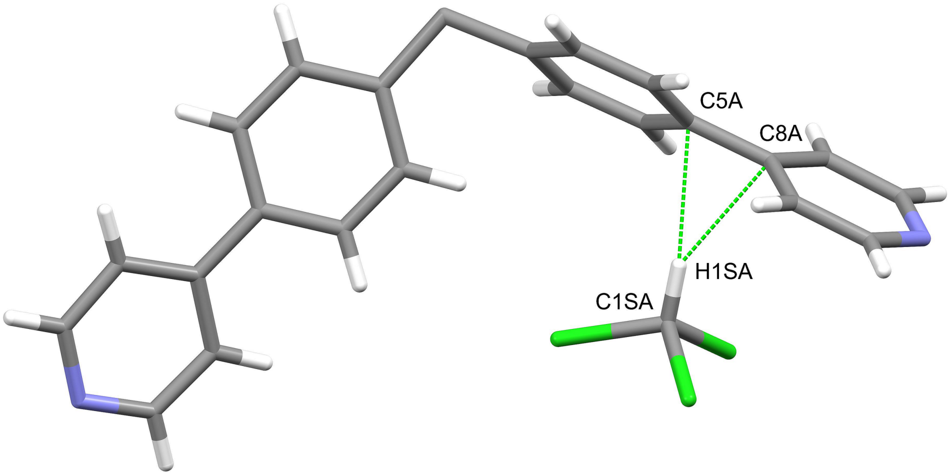

Figure S3. Detail of the C-H···C$_{aromatic}$ interactions in **TPPM·CHCl$_3$**. C1S··C5A and·C1S-H1S···C5A: 3.365(3) Å and 166.9(5)°; C1S··C8A and·C1S-H1S···C8A: 3.209(2) Å and 150.0(6)°.

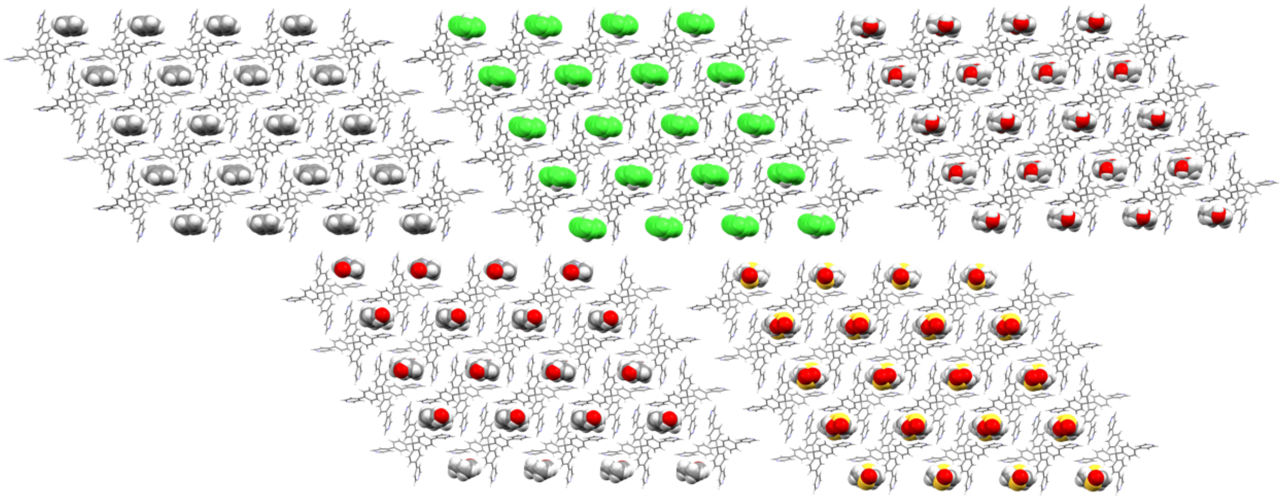

Figure S4. Crystal structure of the different solvates of **TPPM** (from left to right, from top to bottom: C_6H_6, $CHCl_3$, EtOH, DMF, DMSO).

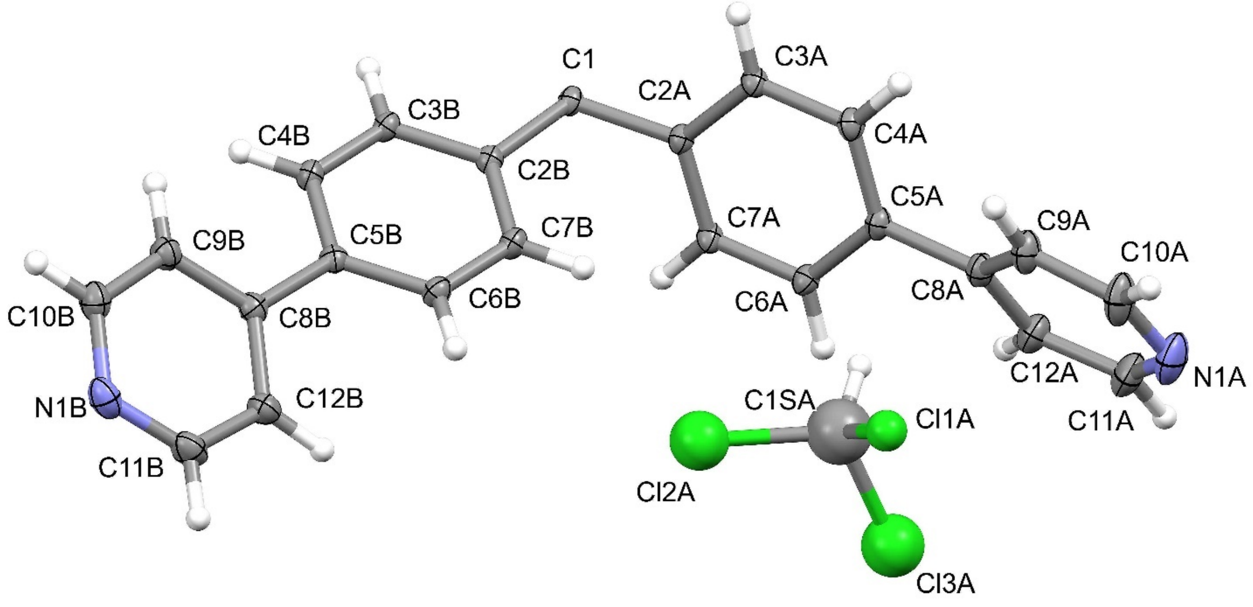

Figure S5. Ortep view of the asymmetric unit of **TPPM·CHCl$_3$**. Only one possible orientation for chloroform is shown for clarity.

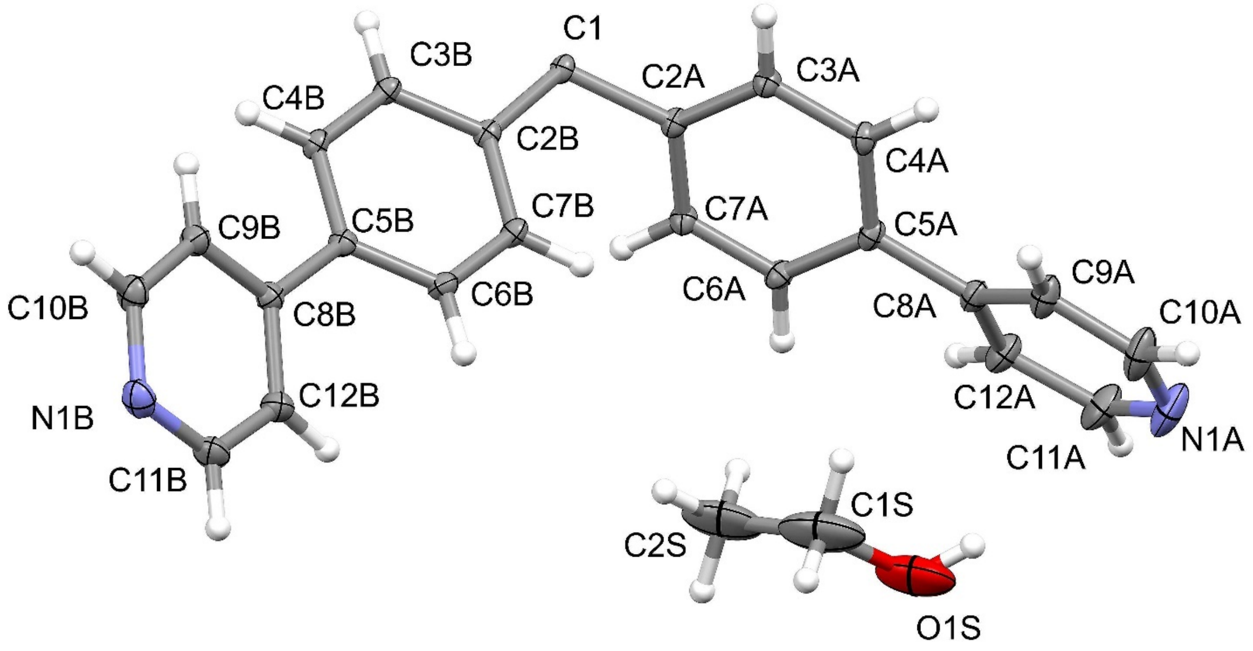

Figure S6. Ortep view of the asymmetric unit of **TPPM·EtOH**. Only one orientation of ethanol is shown for clarity.

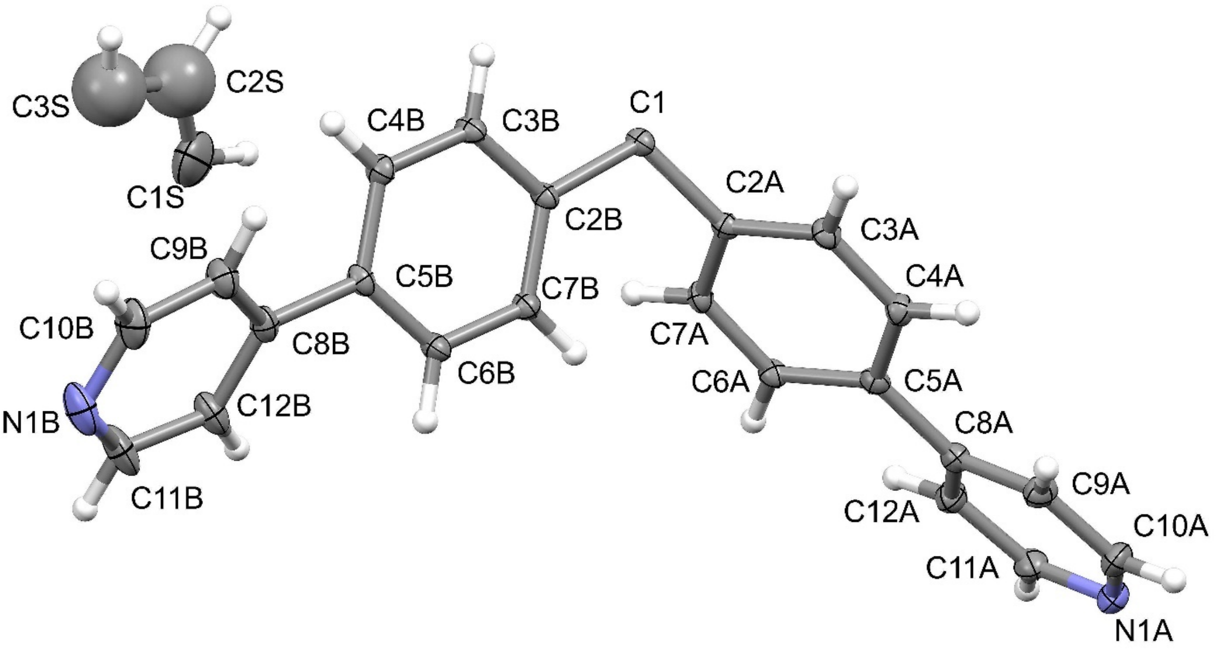

Figure S7. Ortep view of the asymmetric unit of **TPPM·C_6H_6**.

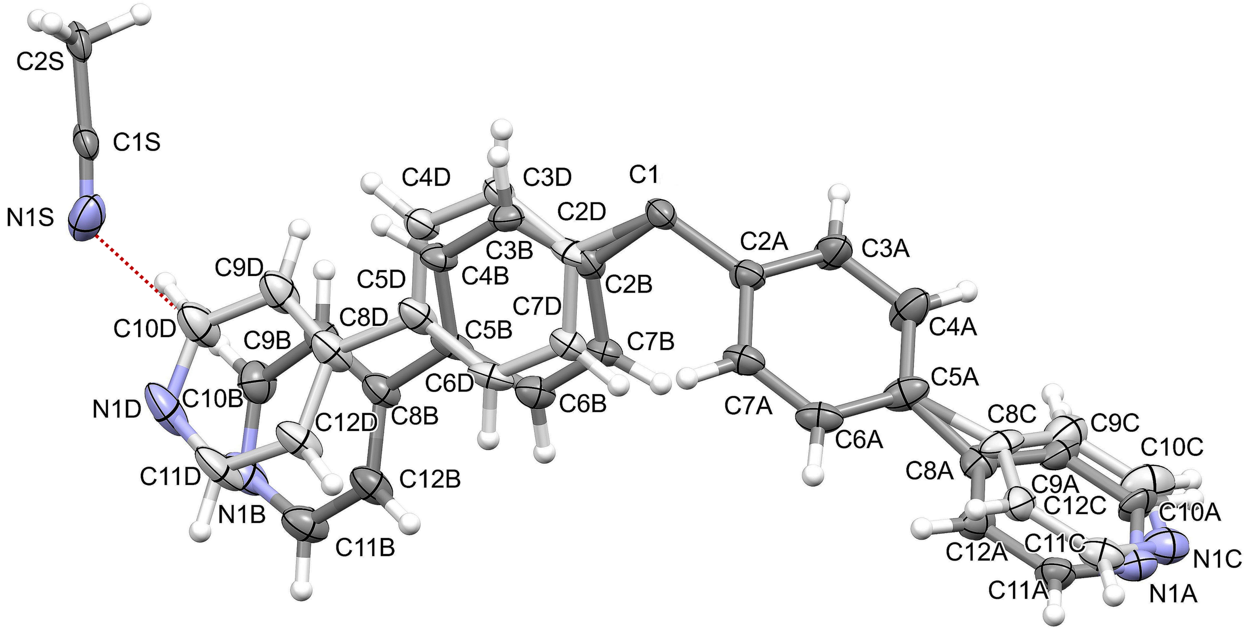

Figure S8. Ortep view of the asymmetric unit of **TPPM·0.5CH$_3$CN.** The second orientation of **TPPM** is shown in light grey. The C10B-H10B···N1S interaction [3.439(3) Å; 123.7(4)°] is shown as a red dotted line.

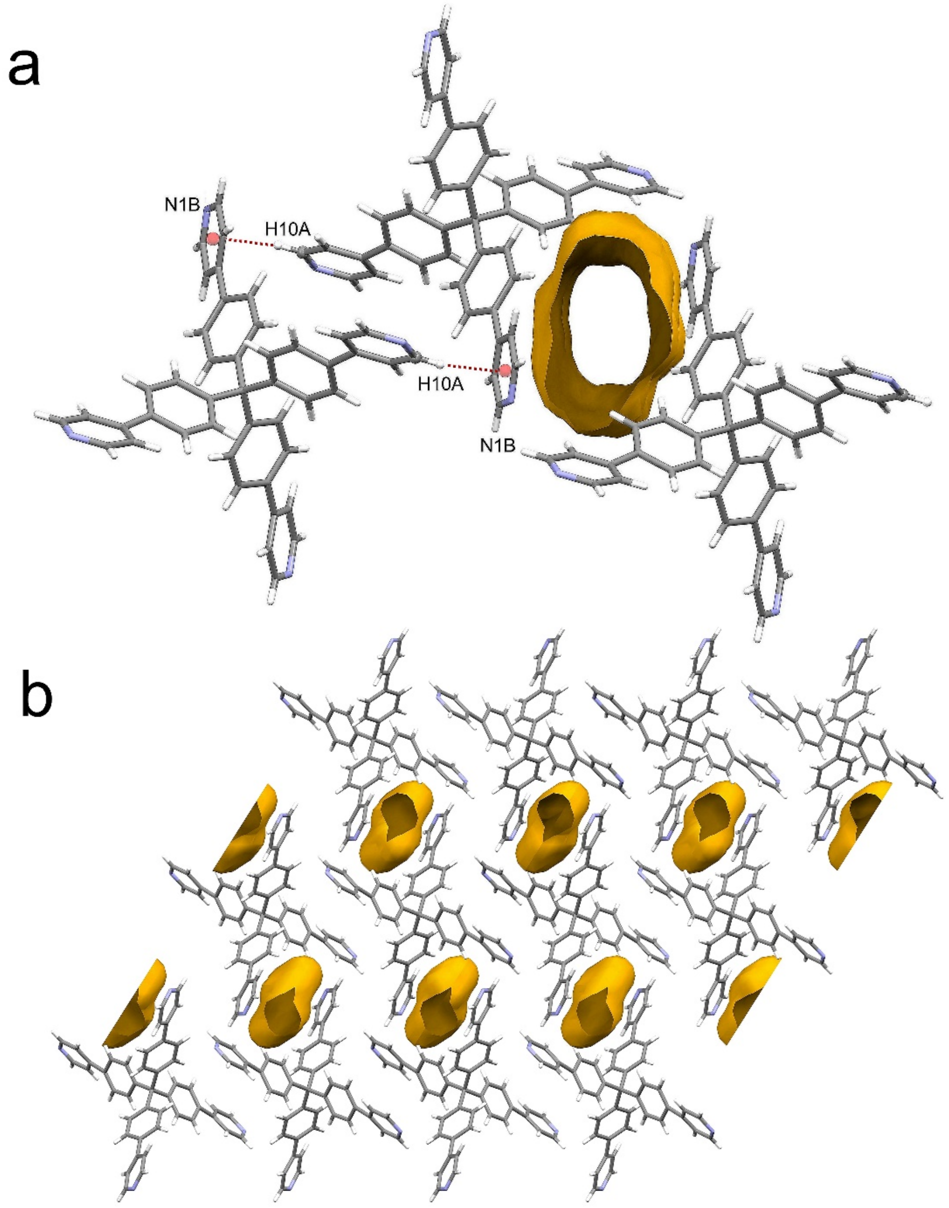

Figure S9. a) View of the C-H···π interactions in **TPPM·0.5CH$_3$CN**. The red spheres are the centroids Cg of ring C8B-C12B/N1B. C10A···Cg: 3.403(3) Å; C10A-H10···Cg, 167.8(5)°. The channels containing the solvent (not shown for clarity) are in yellow. b) View along the *b* axis direction of the empty phase in **TPPM·0.5CH$_3$CN**.

3.2. Hirshfeld surface analysis and fingerprint plots

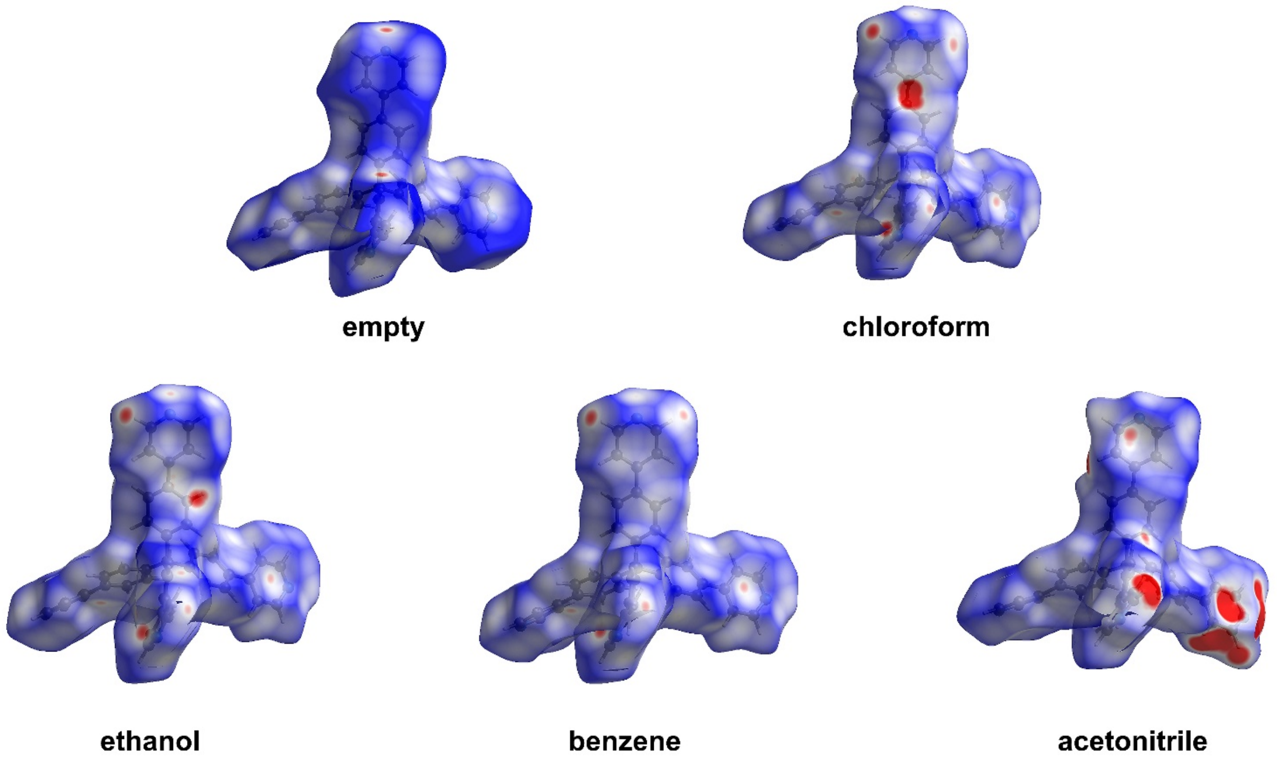

Figure S10. Comparison of the Hirshfeld surface of **TPPM** in the empty form and in **TPPM·CHCl$_3$**, **TPPM·EtOH**, **TPPM·C$_6$H$_6$** and **TPPM·ACN**.

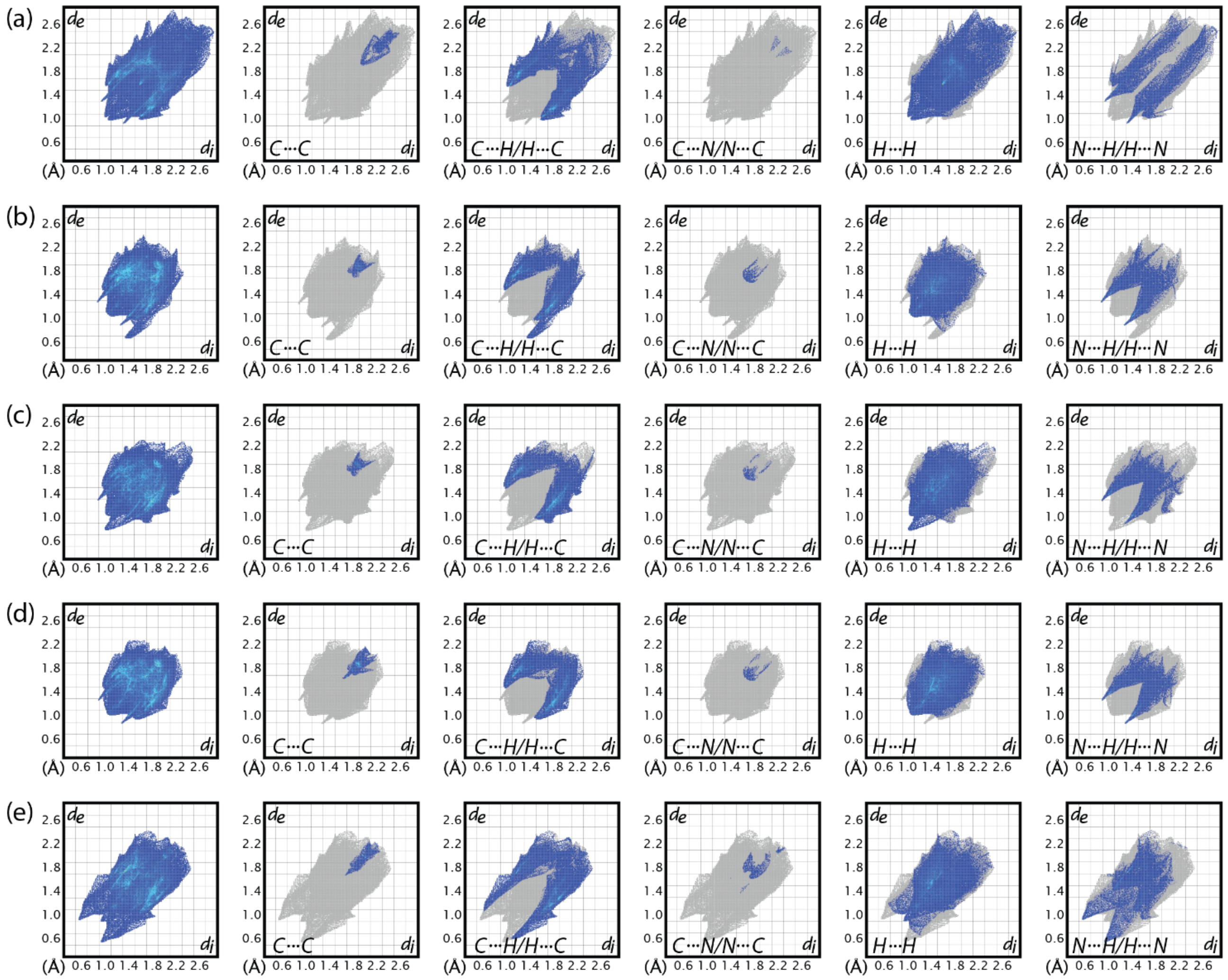

Figure S11. Comparison of the fingerprint plots of (a) **TPPM** in the empty form and in (b) **TPPM·CHCl$_3$**, (c) **TPPM·EtOH**, (d) **TPPM·C$_6$H$_6$** and (e) **TPPM·ACN**.

Table S2. Percentage of the interactions shown in the fingerprint plots.

**Interaction**	**Empty**	**Chloroform**	**Ethanol**	**Benzene**	**ACN-Filled**
C···H/H···C	33.5	28.2	31.3	31.5	33.5
C···C	2.2	2.1	2	4.2	2.6
C···N/N···C	0.1	0.6	0.4	0.5	0.9
N···H/H···N	17.2	14.5	14.6	14.5	18.4
H···H	47	41.8	48.6	49.3	44.6
O···C/C···O	-	-	0.5	-	-
O···H/H···O	-	-	2.6	-	-
C···Cl/Cl···C		4.2	-	-	-
Cl···H/H···Cl		8.3	-	-	-
Cl···N/N···Cl		0.3		-	-

3.3. 3D Electron diffraction (3D ED)

Scanning transmission electron microscopy imaging and 3D electron diffraction have been carried out on a Zeiss Libra 120 transmission electron microscope, equipped with a LaB_6 thermionic source operating at 120 kV (λ=0.0335 Å) and a Timepix single-electron detector by ASI for collecting diffraction patterns in low dose mode. 3D electron diffraction data were collected on single nanocrystals in nanodiffraction mode with a parallel electron beam of 150 nm in diameter. The diffraction patterns were collected in stepwise mode, with an angular step of 1° covering an angular range of 120°. The data collection was carried out in precession mode with a parallel beam precessing on a cone surface with a 1° semiangle aperture (PEDT protocol), in order to increase the reciprocal space integration. Imaging was carried out in STEM mode with a high angular dark field detector (HAADF). Crystal data and experimental details for data collection and structure refinement are reported in Table S1. The 3D ED data were analysed using the software PETS.[8] Ab-initio structure determination of **TPPM** was performed by Standard Direct Methods using the SIR2019 package.[9] Data were initially refined with a fully kinematical approximation, i.e. neglecting dynamical scattering and assuming that I_{hkl} is proportional to $|F_{hkl}|^2$. Least-squares structure refinement was performed with the software SHELXL-2014[4] interfaced with ShelXle.[10] The kinematically refined structure was subsequently refined taking into account the dynamical diffraction theory (multiple scattering) of electrons. The diffraction data were properly integrated for the dynamical refinement using PETS. Dynamical refinement of **TPPM** was carried out with the software suite Jana2006.[11] Crystallographic data for **TPPM** have been deposited with the Cambridge Crystallographic Data Centre as supplementary publication no. 2194031.

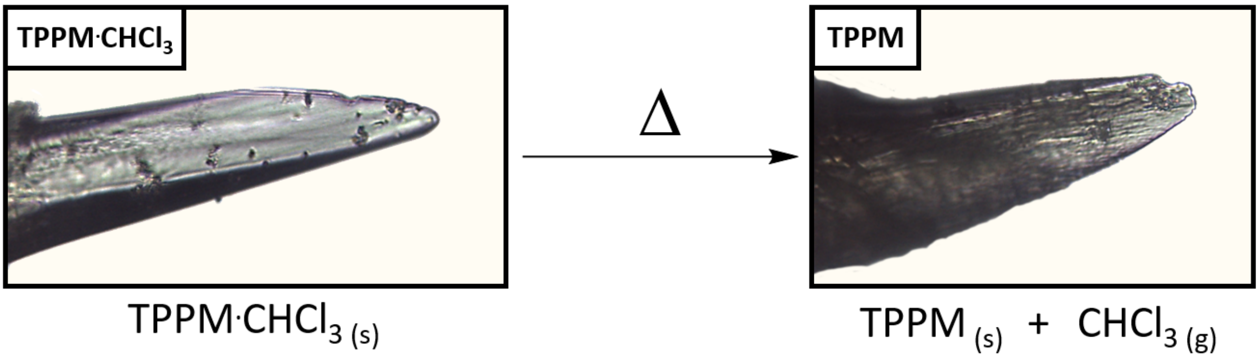

Figure S12. Optical microscope images of a single crystal of **TPPM·CHCl$_3$** before and after thermal activation.

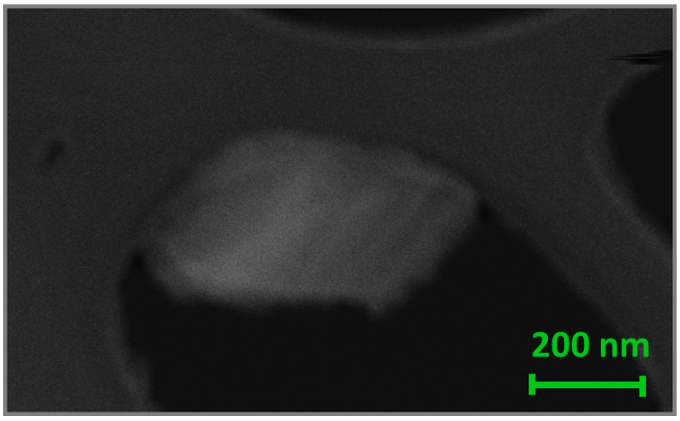

Figure S13. HAADF STEM-image of the **TPPM** microcrystal used for the 3D ED data collection.

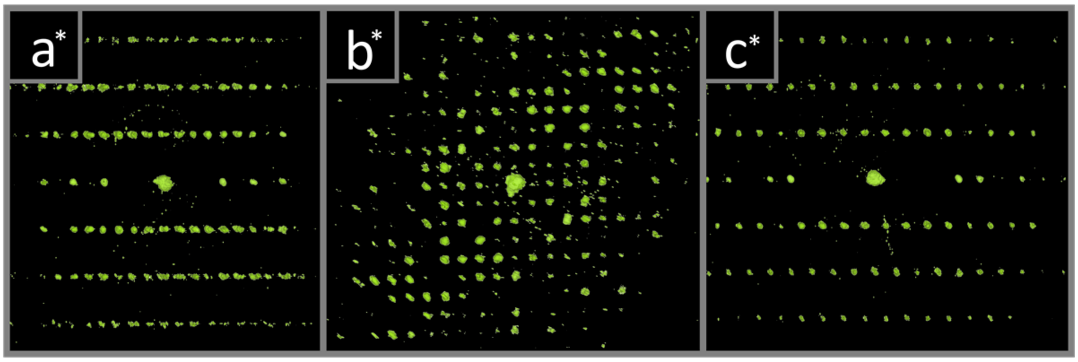

Figure S14. Projection along the three reciprocal cell directions of the reconstructed reciprocal space of **TPPM** on the basis of 3D ED data.

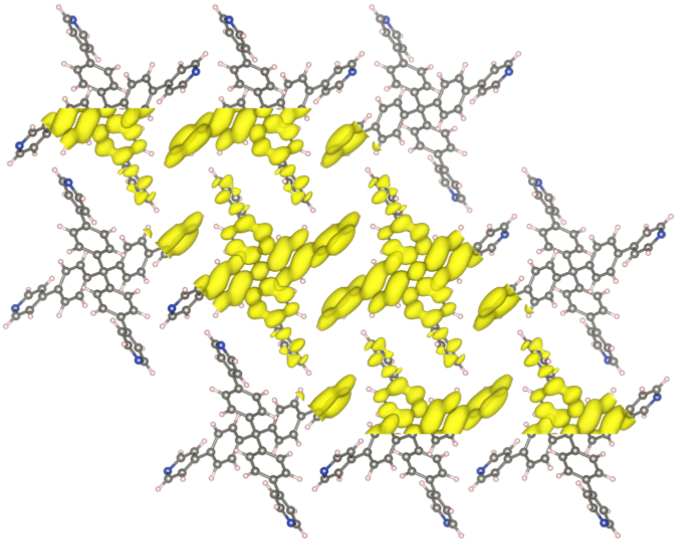

Figure S15. Electrostatic potential map of the **TPPM** structural model, visualized as isosurface within the unit cell border. The potential map is calculated from the dynamically refined structure by Fourier map calculation.

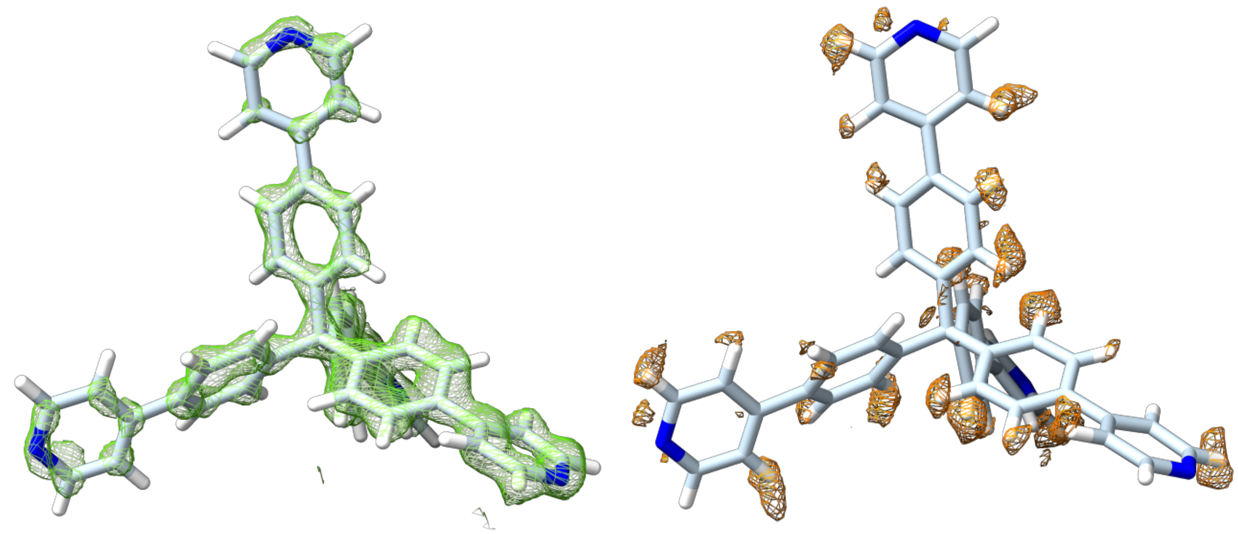

Figure S16. Left: Superposition between the molecular unit and the calculated potential map, in the dynamically refined phase of **TPPM**. Right: Superposition between the difference potential map, calculated from a structural model without H atoms, and the **TPPM** molecular unit.

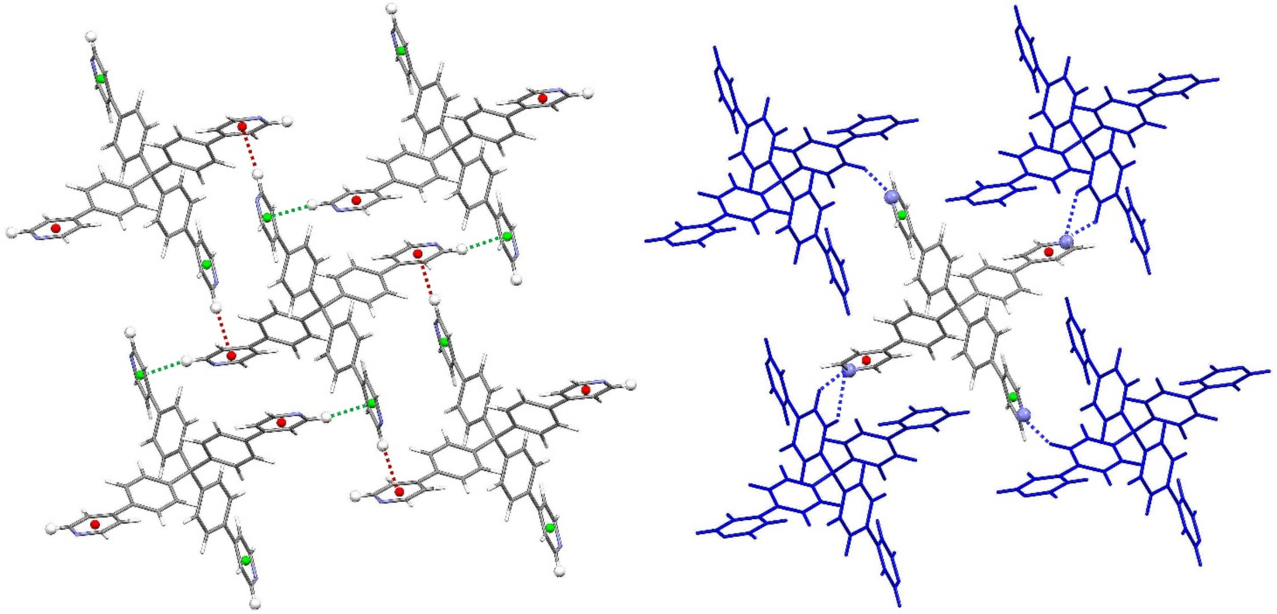

Figure S17. View along the *b*-axis direction of the main C-H···centroids (left) and C-H···N (right) interactions, present in the supramolecular framework of the empty form of **TPPM**. The centroids are represented as green (C8A-C12A/N1A, Cg1) and red (C8B-C12B/N1B, Cg2) spheres, respectively. C11B-H11B···Cg1, 3.770(4) Å and 173.2(3)°. C10A-H10A···Cg2, 3.984(5) Å and 165.2(6)°. C6B-H6B···N1A, 3.357(3) Å and 141.3(4)°. C3A-H3A···N1B, 3.462(4) Å and 119.3(5)°. C4A-H4A···N1B, 3.370(2) Å and 126.5(6)°.

3.4. *Powder X-ray diffraction (PXRD)*

The PXRD patterns of the samples were collected using Ni-filtered Cu K□ radiation ($\lambda_{K\alpha1}$ = 1.5406 Å, $\lambda_{K\alpha2}$ = 1.5444 Å), on a Rigaku SmartLab XE diffractometer equipped with a HyPix-3000 detector. The data were preliminarily processed with SmartLab Studio II (by Rigaku). Standard PXRD patterns were collected in Bragg-Brentano geometry in the 2θ range 5-35°, placing the sample on a silicon zero background specimen holder. Temperature-resolved *in situ* data collections were performed using a TTK 600 temperature chamber by Anton Paar, collecting each pattern in a 2θ range of 5-27° in 5 minutes during the heating ramp (1°C min^{-1}).

The LeBail refinement on powder X-ray diffraction data, from temperature-resolved analysis, was carried out with Jana2006.[11] The background was described by manually-picked points, the unit cells were defined from single crystal data (SC-XRD or 3D ED) and the profile parameters were obtained by cyclic refinements on the entire dataset. The convergence residual values of each diffraction pattern are reported in Table S3. The diffraction profile temperature is the mean value between initial and final data collection temperatures. Each diffractogram, collected during the temperature resolved experiment, was analyzed through LeBail refinement. The profile data obtained from the refinement were used for the phase fraction estimation of **TPPM·S** (**TPPM·$CHCl_3$**) and empty **TPPM**. For both phases, a characteristic reflection was selected: (-202) for **TPPM** at 2θ = 8.02° and (111) for **TPPM·S** at 2θ = 14.39°. The phase fraction calculation was performed comparing the peak area of the characteristic reflection for each powder diffraction pattern, in accordance with Equation S1.

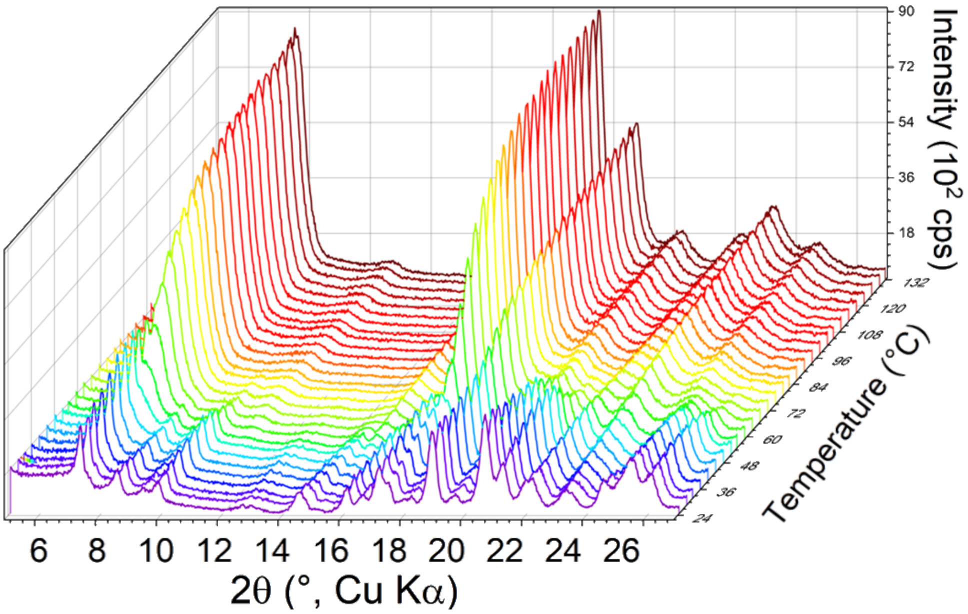

Figure S18. X-ray powder profile collected during the temperature-resolved analysis.

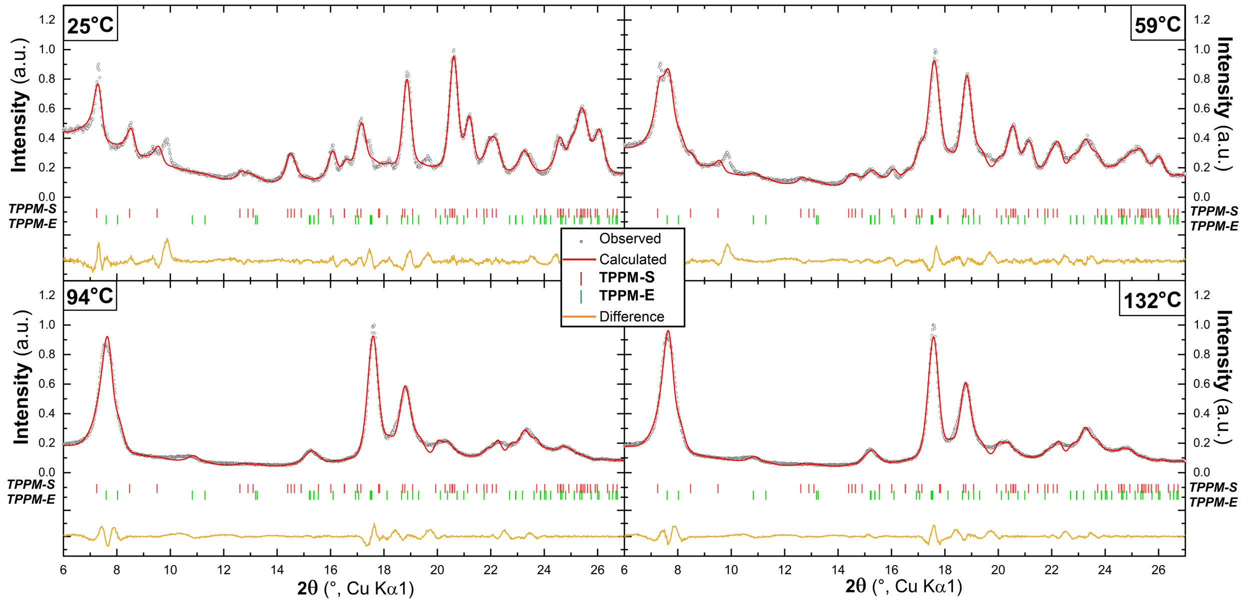

Figure S19. Profile fit from LeBail refinement performed on specific diffractograms at characteristic temperatures of the PXRD desorption monitoring experiment. The convergence residual values are reported in Table S3.

$$\chi_{TPPM}(T) = \frac{A_{TPPM}(T)}{A_{TPPM}(T) + A_{TPPM\cdot S}(T)} \cdot 100$$

Equation S1. Equation used for the calculation of the **TPPM** phase fraction (χ_{TPPM}). $\mathbf{A_{TPPM}(T)}$ and $\mathbf{A_{TPPM\cdot S}(T)}$ are the areas of the (-202) and (111) reflections, respectively, for **TPPM** and **TPPM·CHCl$_3$** at the temperature T.

Table S3. Profile parameters for each diffractogram collected during the temperature-resolved experiment.

Temperature (°C)	R_p (%)	R_{wp} (%)
21	5.4844	7.9947
25	5.4121	8.0852
29	5.5843	8.3577
32	5.7252	8.8281
36	5.7962	9.1395
40	6.1616	9.7719
44	6.0805	10.053
48	5.9376	9.7978
52	5.4259	9.2085
55	5.1261	8.2017
59	5.0332	7.3885
63	5.0167	6.9963
67	5.1857	7.0132
71	5.4371	7.4023
75	5.4544	7.346
79	5.4411	7.3796
82	5.4497	7.3435
86	5.5465	7.4895
90	5.4314	7.2961
94	5.3235	7.2297
98	5.3435	7.1588
102	5.2542	7.1246
105	5.2839	7.1011
109	5.1544	6.9531
113	5.2909	7.008
117	5.3109	7.0219
121	5.1686	6.8925
125	5.2629	6.9829
129	5.2704	6.8826
132	5.2591	6.9453

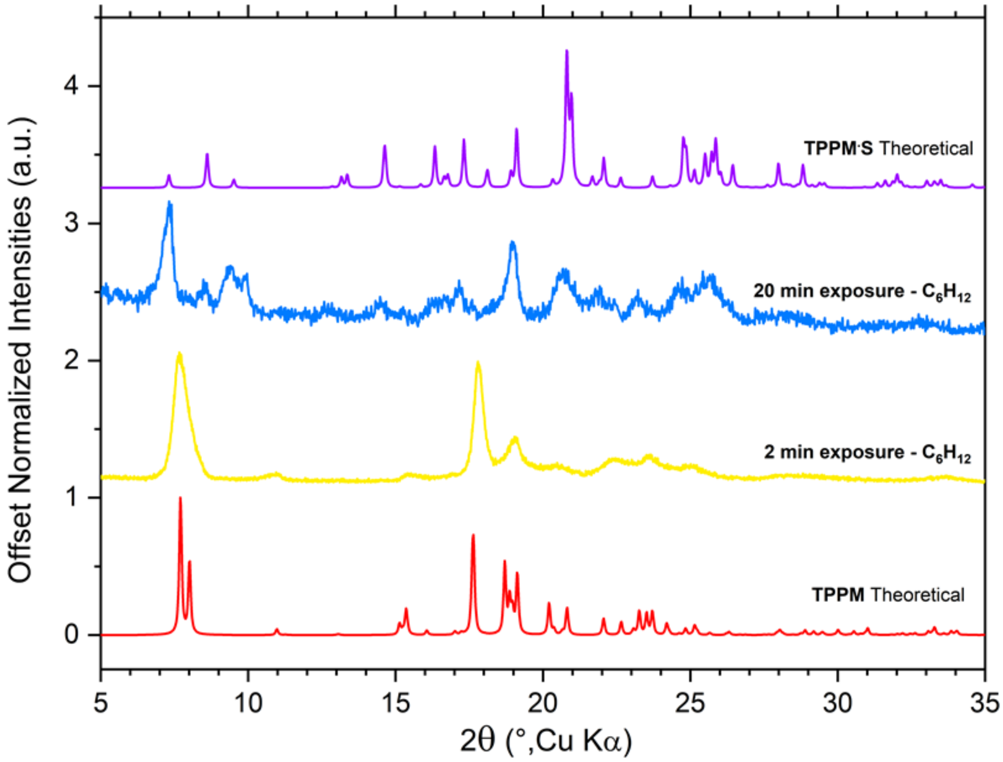

Figure S20. Powder X-ray diffractograms of **TPPM** after 2 min and 20 min of exposure to cyclohexane vapors.

4. Thermal characterization

4.1. Differential Scanning Calorimetry (DSC)

Differential scanning calorimetry analyses were performed with a Perking Elmer instrument, model DSC6000. The desorption analyses were performed in holed pans with a heating rate of 5°C min^{-1} in a temperature range of 30-220 °C. The cyclic absorption-desorption experiment was performed in a closed pan with a heating rate of 10 °C min^{-1} in a temperature range of 60-200°C.

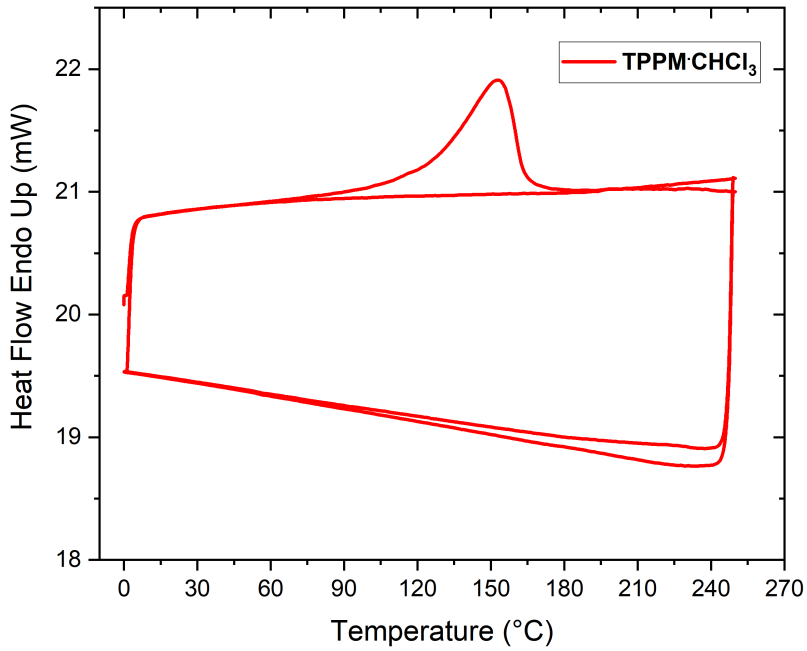

Figure S21. DSC thermogram of the **TPPM·$CHCl_3$** phase, performed with two heating-cooling cycles to highlight the stability of the **TPPM** phase after solvent removal.

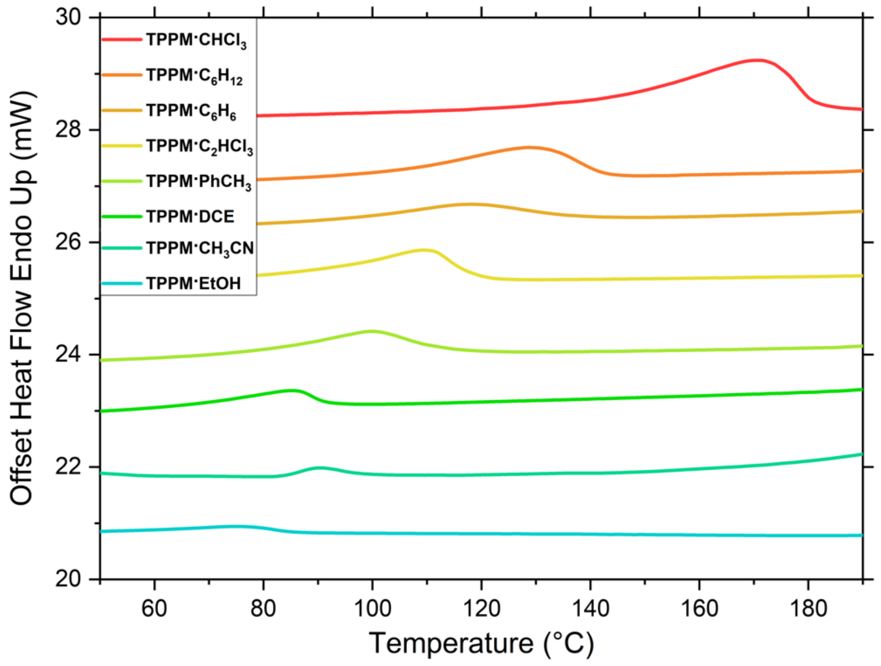

Figure S22. Comparison of the thermograms of various **TPPM** solvates highlighting the different heats of desorption; only the heating ramps have been reported. The dichloroethane solvate (**TPPM·DCE**) presents an intermediate value of ΔH_{Des} that can probably be related to its low pore occupancy.

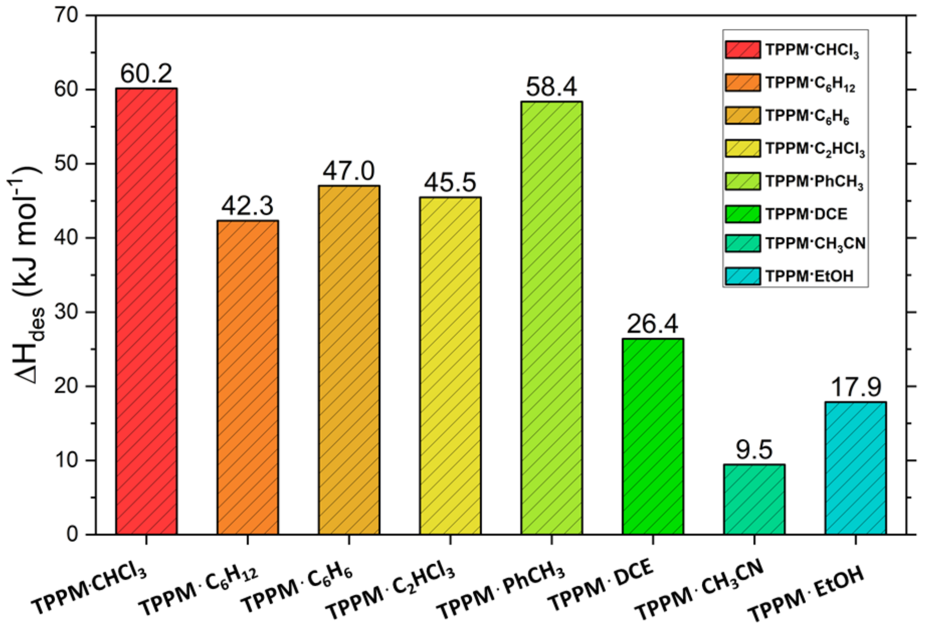

Figure S23. Desorption Enthalpies derived from DSC characterization on different **TPPM·S** phases.

4.2. *Thermogravimetric Analysis (TGA)*

Thermogravimetric analyses were performed with a Perking Elmer instrument, model TGA 8000. The desorption process leading to the weight loss was performed under a heating rate of 5°C min^{-1} in a temperature range of 30-220 °C.

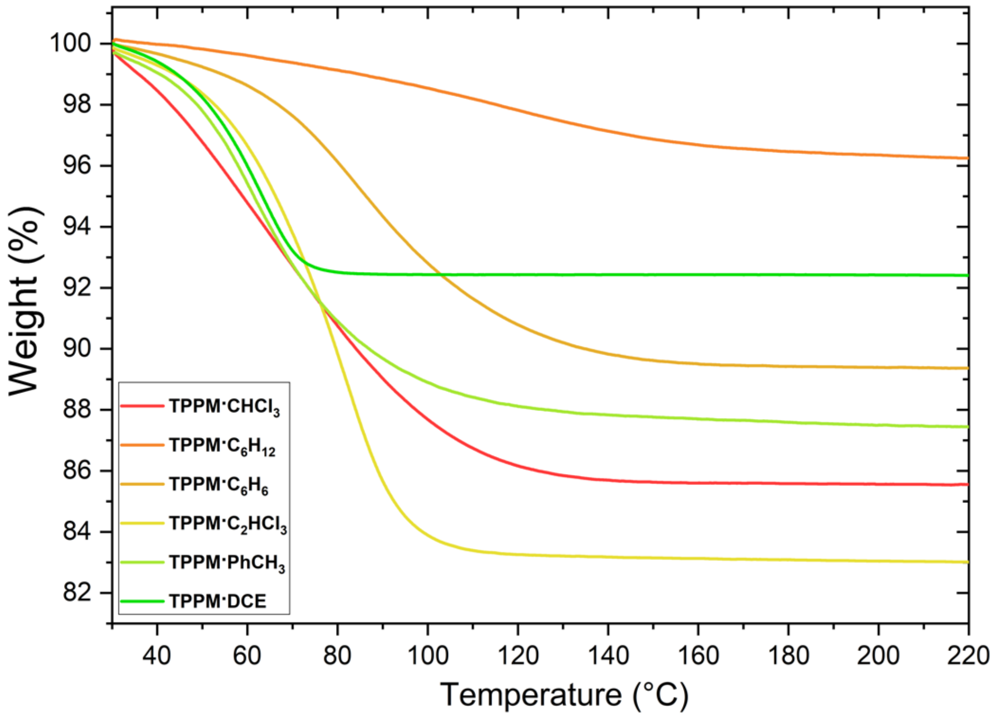

Figure S24. Comparison of the thermogravimetric path for different **TPPM** solvates

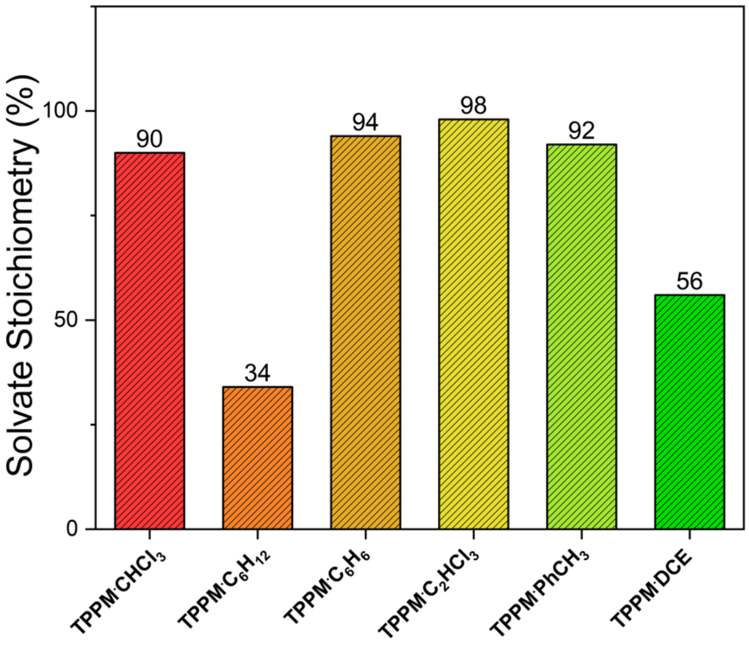

Figure S25. Comparison of the solvate stoichiometry among the **TPPM** solvates. The solvate stoichiometry (SS^{TGA}) was estimated from TGA data using Equation S3.

$$SS^{TGA} = \frac{\Delta W(\%) \cdot (MW_{TPPM} + MW_{Guest})}{MW_{Guest}}$$

Equation S3. Relation adopted for the solvate stoichiometry (SS^{TGA}) calculation from TGA data.

5. Packing coefficients calculation

The packing coefficients were calculated as the ratio between the guest volume (V_{Guest}) and the volume of the guest-binding site (V) present in the **TPPM·S** phase (Equation S2). Guest volumes were calculated combining the van der Waals Volume (v_{W}) with the probe (radius 1.2 Å) excluded volume (v_{Pex}), using the MoloVol program.[12] The guest binding site volume in the **TPPM·S** phase was calculated dividing the potential void volume by the Z value of the crystal structures. The **TPPM·S** potential void volume was calculated by the program Mercury[13], using a probe with a radius of 1.2 Å, considering the frameworks of the **TPPM·S** crystal structures from which the solvent molecules were removed (15.5 % of the unit cell, 565 $Å^{-3}$).

$$PC_{guest} = \frac{V_{guest}}{V} \cdot 100$$

Equation S2. Relation adopted for the calculation of the guest packing coefficient (PC_{guest}). Guest volume: $V_{\text{guest}} = v_{\text{W}} + v_{\text{Pex}}$; Volume of guest-binding site: $V = 140.5$ $Å^{-3}$.

Table S4. Solvents volumes and Packing Coefficients.

Solvent	v_{W} ($Å^{-3}$)	v_{Pex} ($Å^{-3}$)	V_{guest} ($Å^{-3}$)	PC_{guest} (%)
$CHCl_3$	76.23	1.83	78.06	55.56
DCE	76.78	2.01	78.79	56.08
$PhCH_3$	99.92	1.70	101.62	72.33
C_6H_6	84.03	0.53	84.56	60.18
C_6H_{12}	97.15	3.16	100.31	71.39
CH_3CN	48.34	0.29	48.63	34.61
CH_2Cl_2	59.72	0.94	60.67	42.50
Acetone	62.53	1.72	64.25	44.50
EtOH	52.28	1.12	53.40	38.01
C_2HCl_3	87.78	2.01	89.79	63.91

6. NMR Characterization

The ^{1}H NMR spectra were collected in CD_2Cl_2 with few drops of methanol-d_4, in order to completely solubilize the TPPM molecule, on a Bruker AC400 Avance.

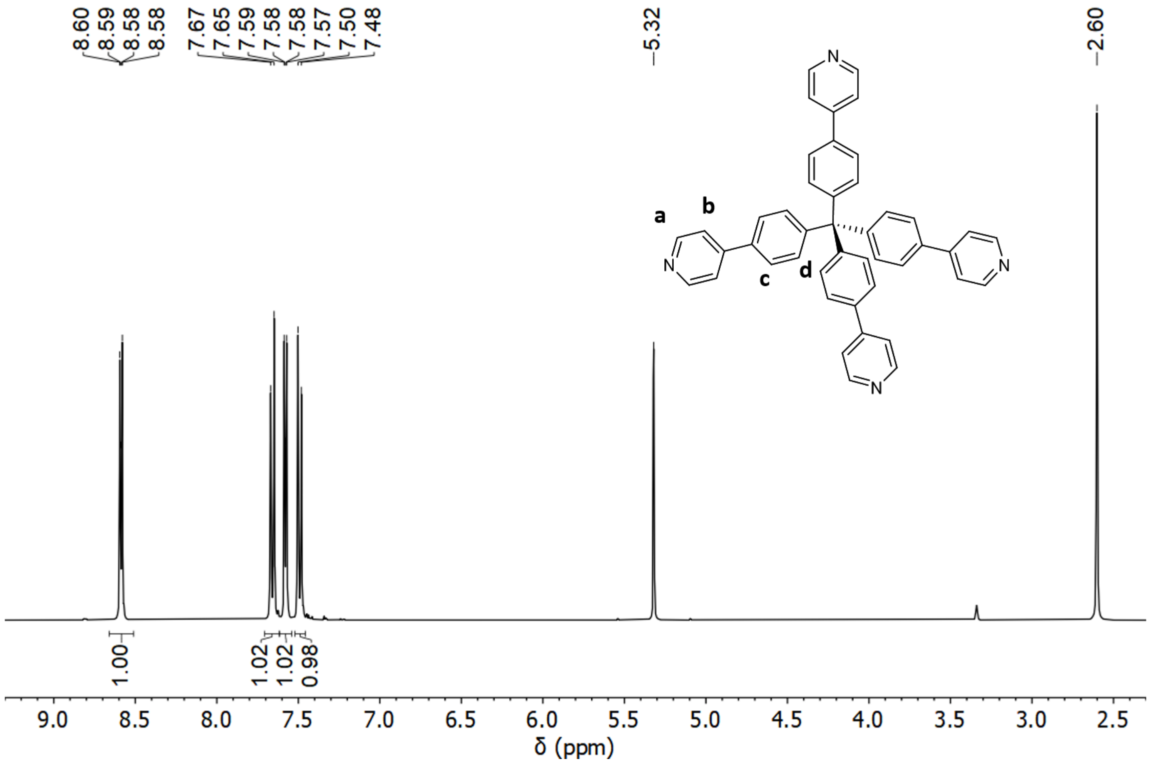

Figure S26. ^{1}H NMR spectrum of **TPPM** (400 MHz, CD_2Cl_2) δ (ppm): 8.59 (dd; J_1 = 4.7 Hz, J_2 = 1.85 Hz, 8H, a), 7.66 (d, J = 8.6 Hz, 8H, c), 7.58 (dd, J1 = 4.7 Hz, J2 = 1.8 Hz, 8H, b), 7.49 (d, J = 8.5 Hz, 8H, d).

*6.1. Solvent loading determination by 1H NMR spectroscopy performed on **TPPM·S** phases.*

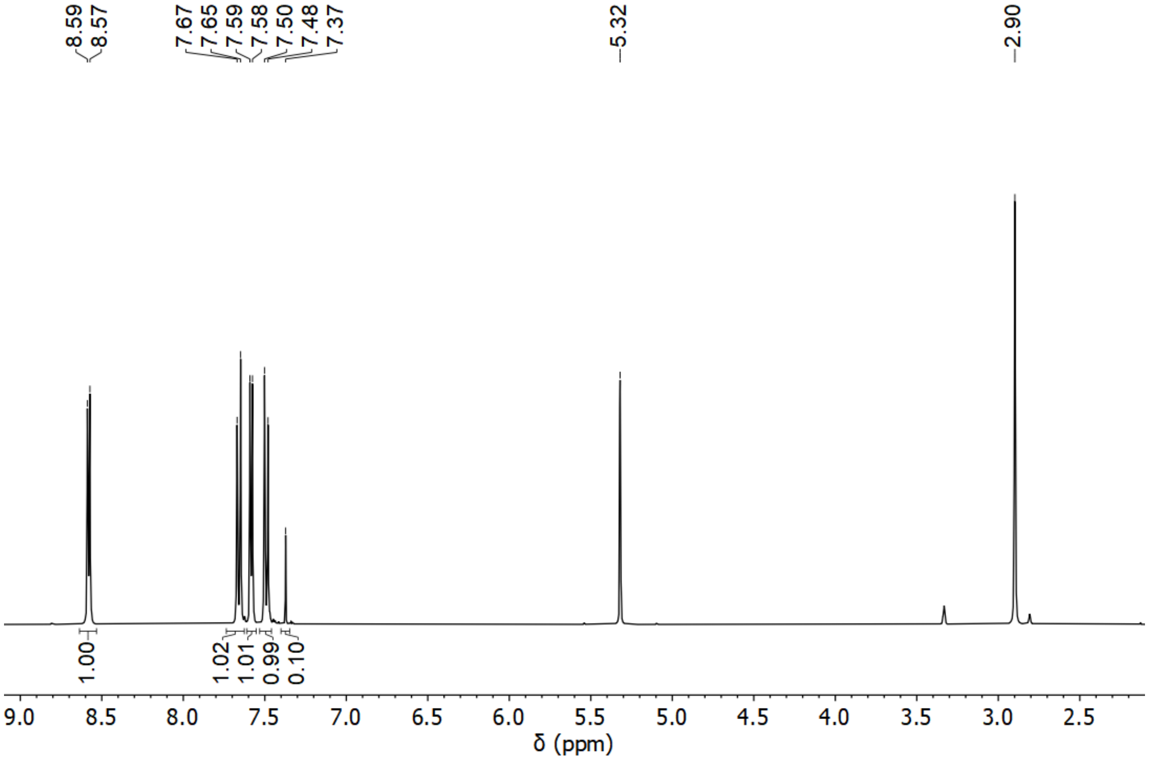

Figure S27. ^{1}H NMR spectrum of **TPPM·CHCl$_3$** collected in CD_2Cl_2.

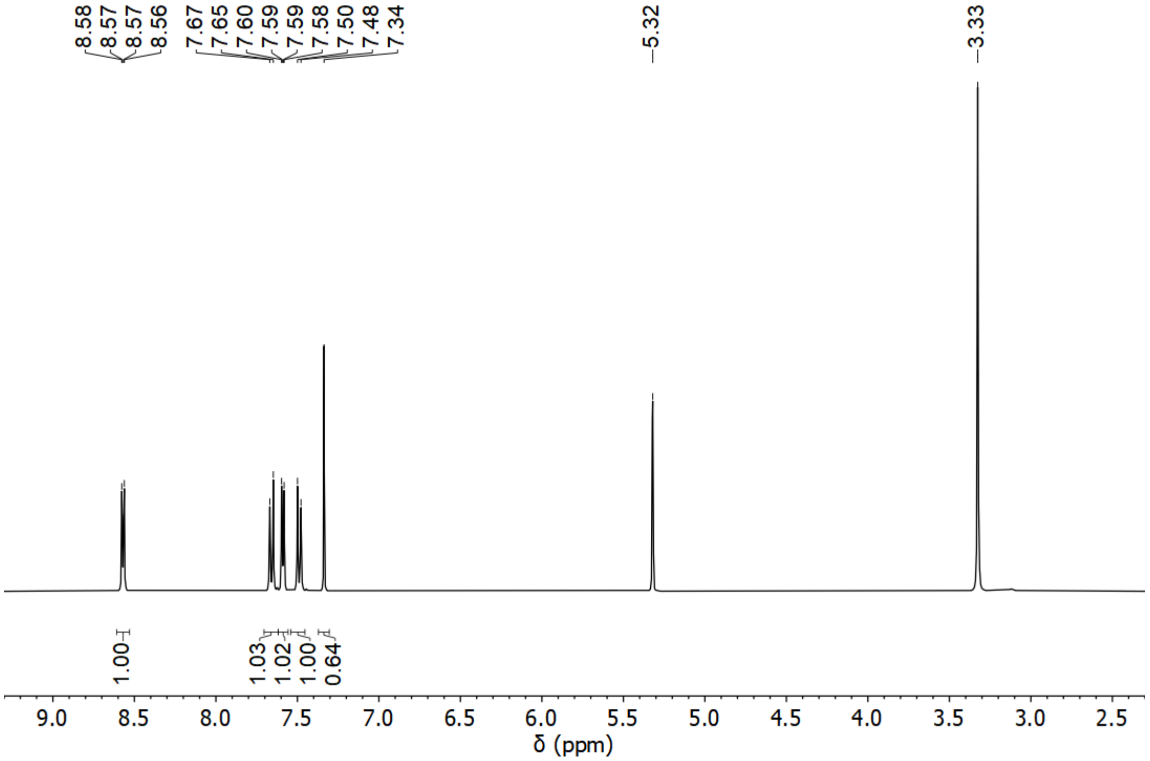

Figure S28. ^{1}H NMR spectrum of **TPPM·C$_6$H$_6$** collected in CD_2Cl_2.

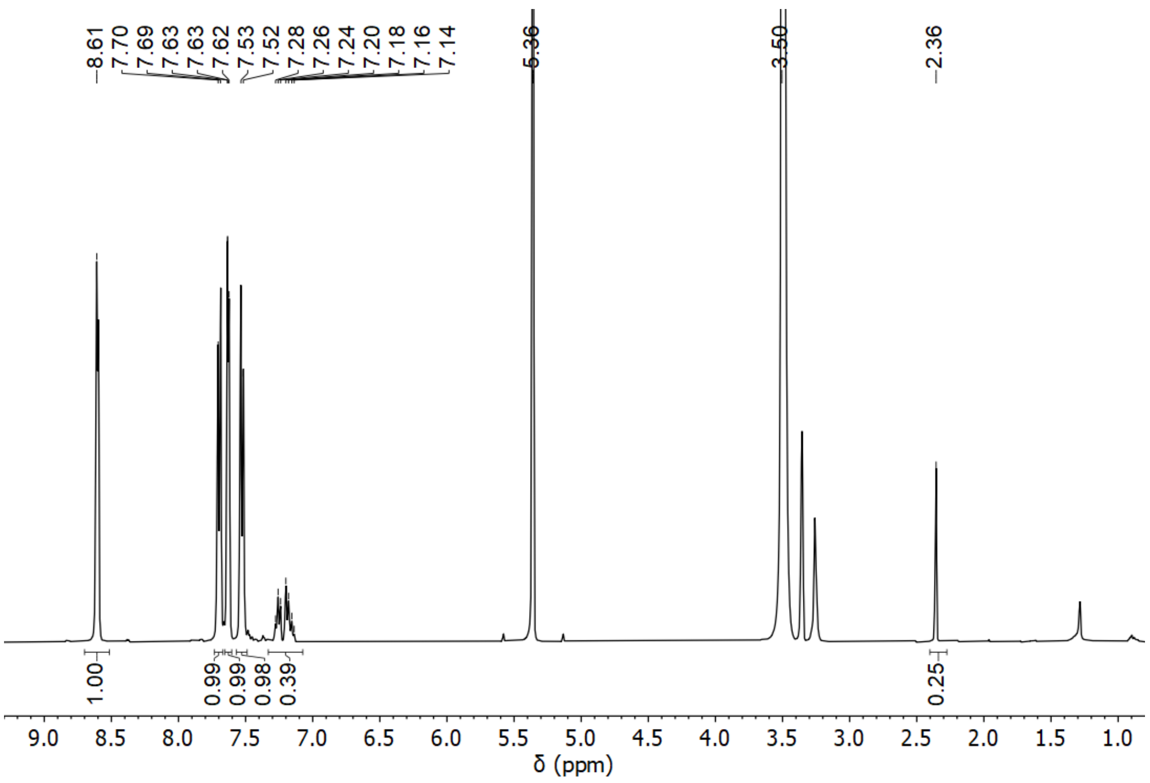

Figure S29. ^{1}H NMR spectrum of **TPPM·PhCH$_3$** collected in CD_2Cl_2.

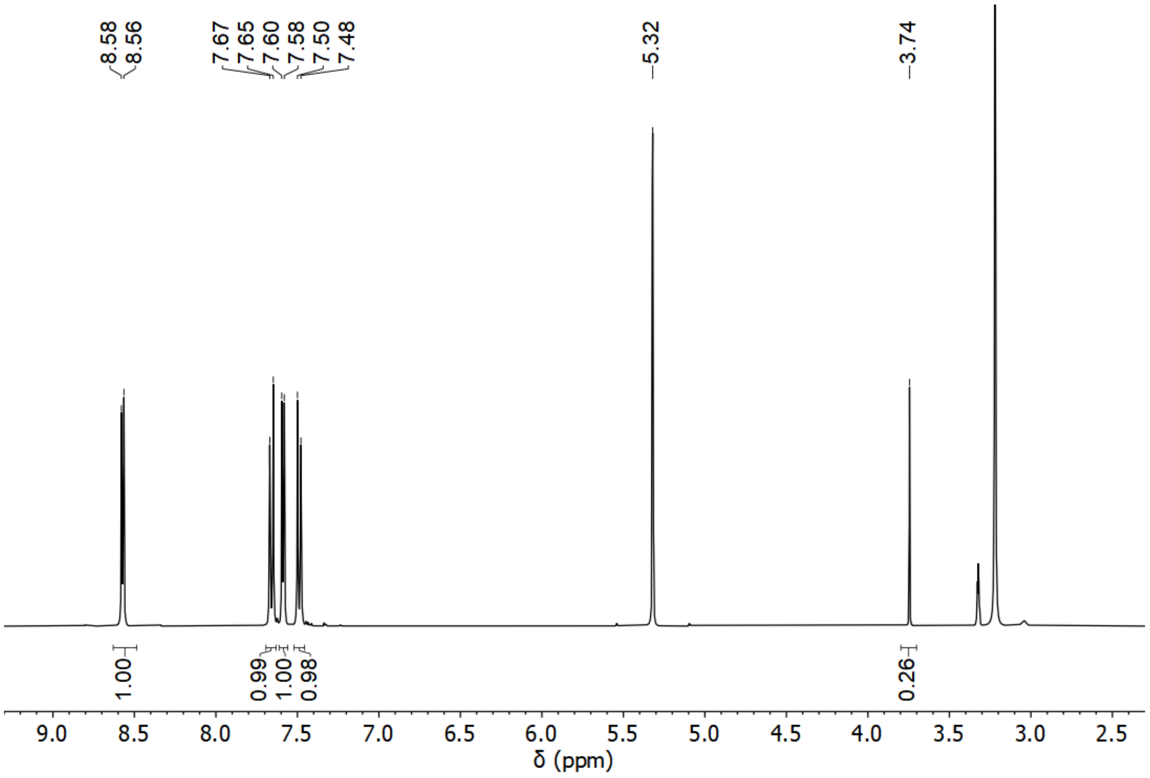

Figure S30. ^{1}H NMR spectrum of **TPPM·DCE** collected in CD_2Cl_2.

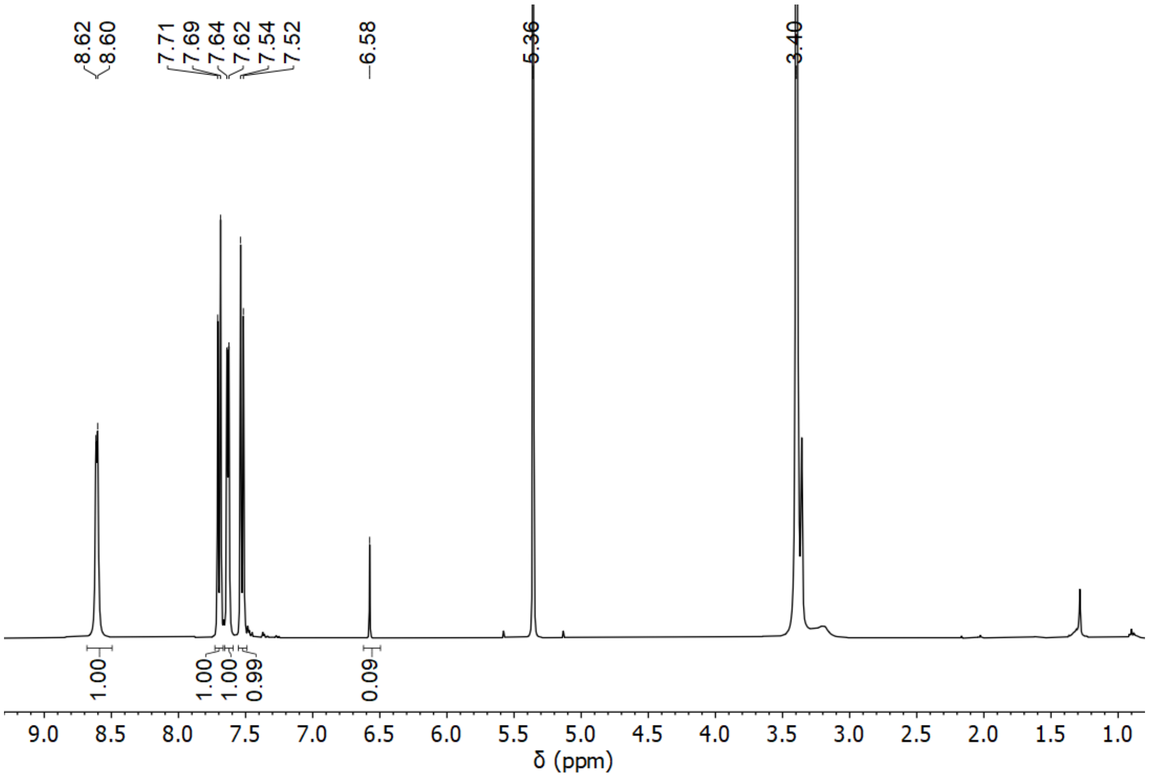

Figure S31. ^{1}H NMR spectrum of **TPPM·C_2HCl_3**solvate collected in CD_2Cl_2.

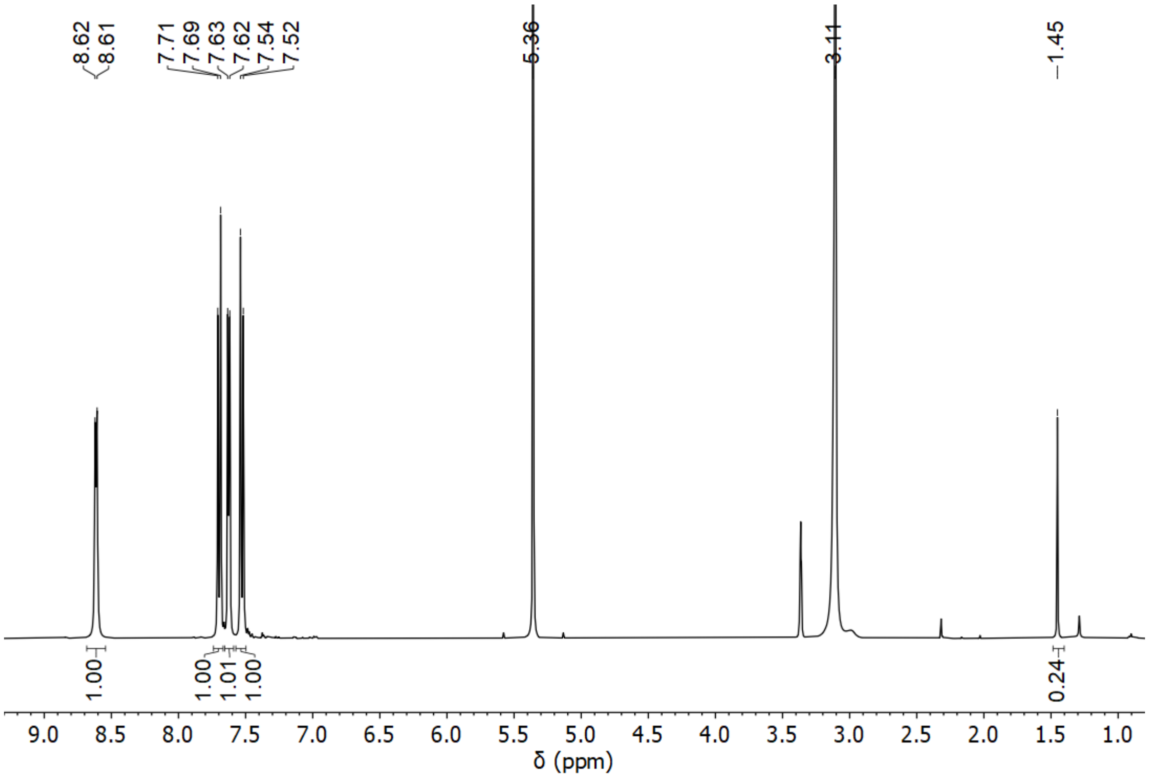

Figure S32. ^{1}H NMR spectrum of **TPPM·$C_{12}H_{12}$** collected in CD_2Cl_2.

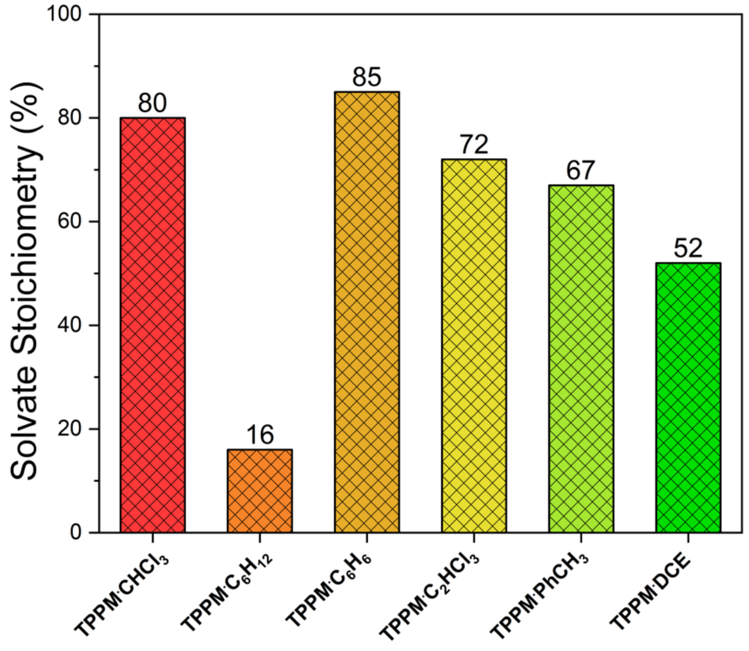

Figure S33. Comparison of the solvate stoichiometry among the different **TPPM** solvates. The solvate stoichiometry (SS^{NMR}) was estimated from ^{1}H NMR data according to Equation S4.

$$SS^{NMR} = \frac{I_{Guest} \cdot n_{TPPM}}{I_{TPPM} \cdot n_{Guest}}$$

Equation S4. Equation adopted for the SS^{NMR} calculation from NMR data. I_{Guest} : integral associated to a guest diagnostic peak; I_{TPPM} : integral associated to a **TPPM** diagnostic peak; n_{Guest} : number of hydrogen atoms related to the guest diagnostic peak; n_{TPPM} : number of hydrogen atoms related to the **TPPM** diagnostic peak

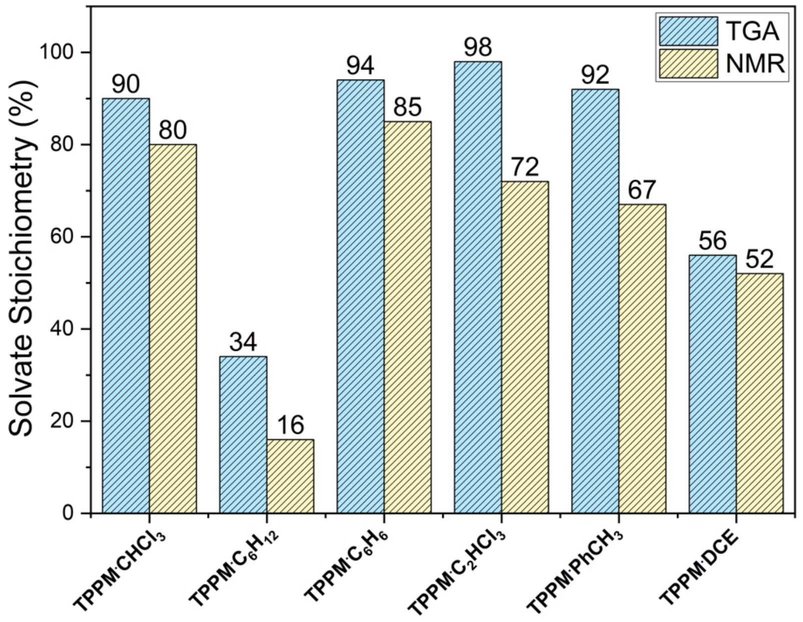

Figure S34. Comparison of SS^{TGA} and SS^{NMR} for different **TPPM** solvates.

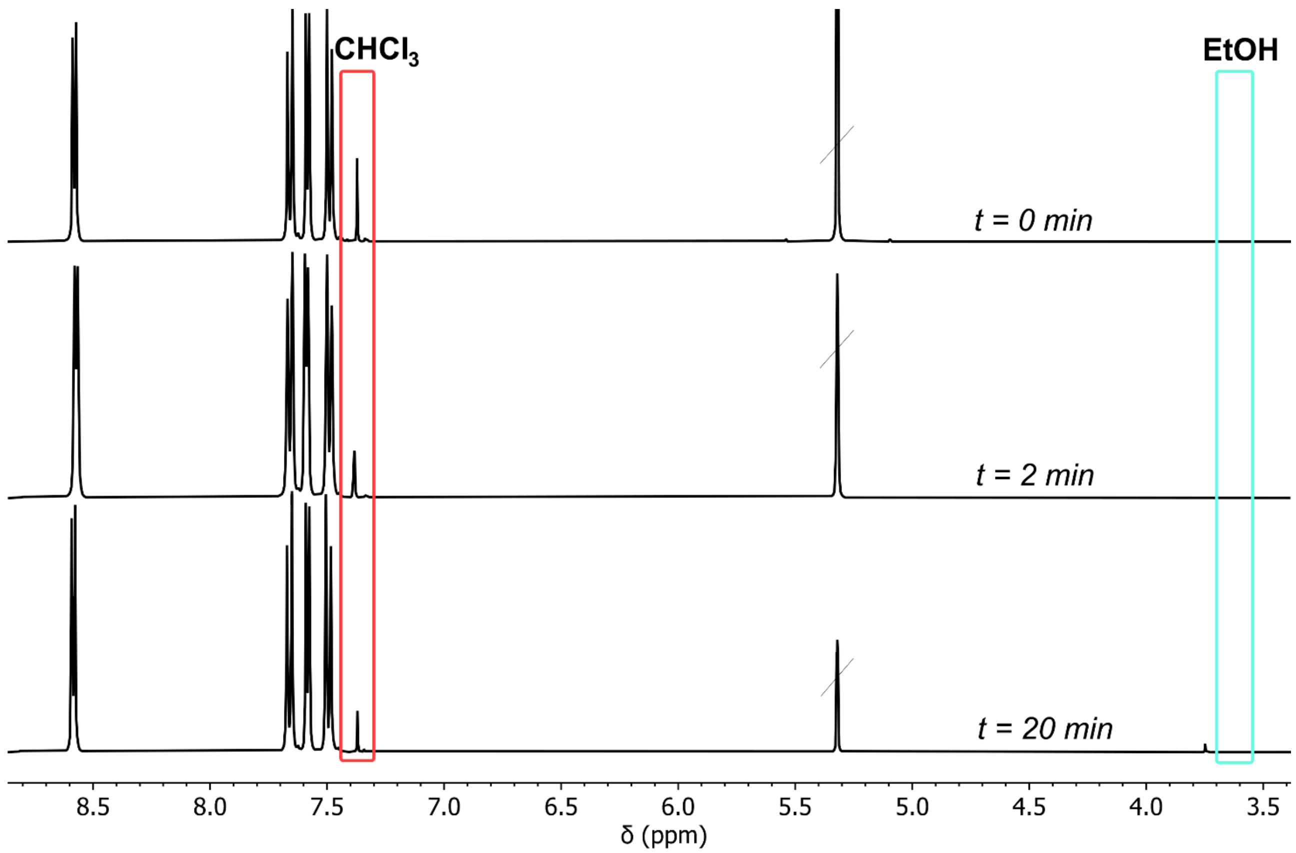

Figure S35. Solvent competition studies followed by ^{1}H NMR spectroscopy. The **TPPM·CHCl$_3$** phase was exposed to EtOH vapours for 2 and 20 minutes. The solid was then dissolved in CD_2Cl_2 with few drops of methanol-d$_4$. ^{1}H NMR spectra were collected and compared to the one of the initial solvate.

7. Spectroscopic Characterization

UV-Vis (UltraViolet-Visible) absorption spectra were collected using a PerkinElmer Lambda650 spectrophotometer. The solid samples were prepared spreading the sample on a quartz plate to produce a thin layer. Absorption spectra were collected in transmission, with the light beam orthogonal to the sample, using air as reference. Liquid samples were analyzed as diluted solution ($\approx 10^{-6}$) in DCM:MeOH (95:5).

Fluorescence measurements were performed on a FLS1000 Edinburgh Fluorometer; the samples were prepared in the same way of the UV-Vis analysis. Emission spectra on solid samples were collected on thinner layers with respect to the absorption spectra, to minimize the inner-filter effects and to obtain an excitation profile comparable to the absorption spectra. Fluorescence analyses were performed placing the quartz support at 45° with respect to the excitation beam and tilted off the vertical. Furthermore, long pass filters were employed in the emission path ($\lambda_{cut-off}$[emission] = 330 nm, $\lambda_{cut-off}$[excitation] = 455 nm).

The solid-state spectroscopic characterization was performed on the **TPPM·S** phases directly obtained by vapor absorption from the empty **TPPM** phase.

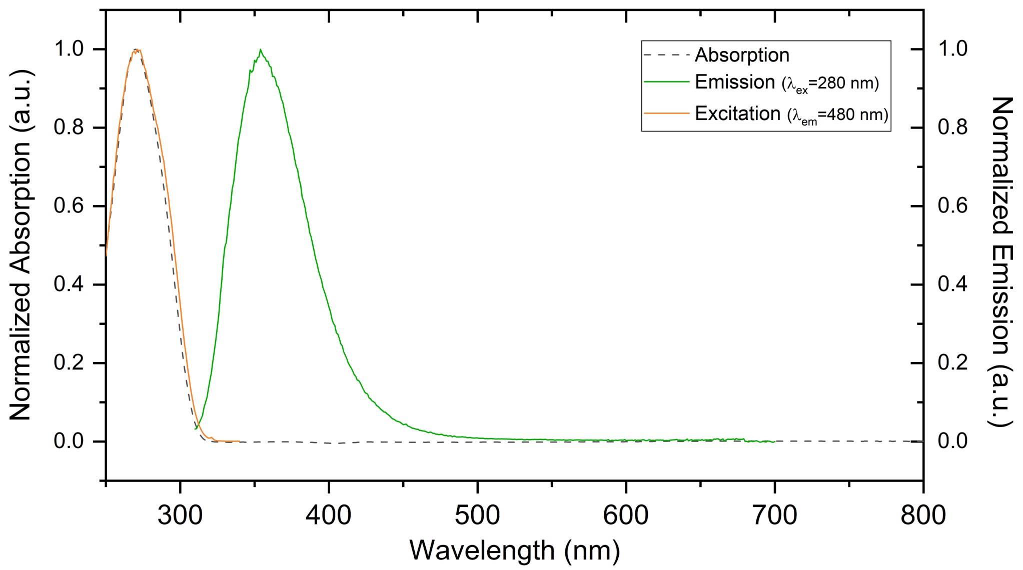

Figure S36. Absorption, excitation, and emission spectra of the empty **TPPM** phase in DCM:MeOH (95:5) solution.

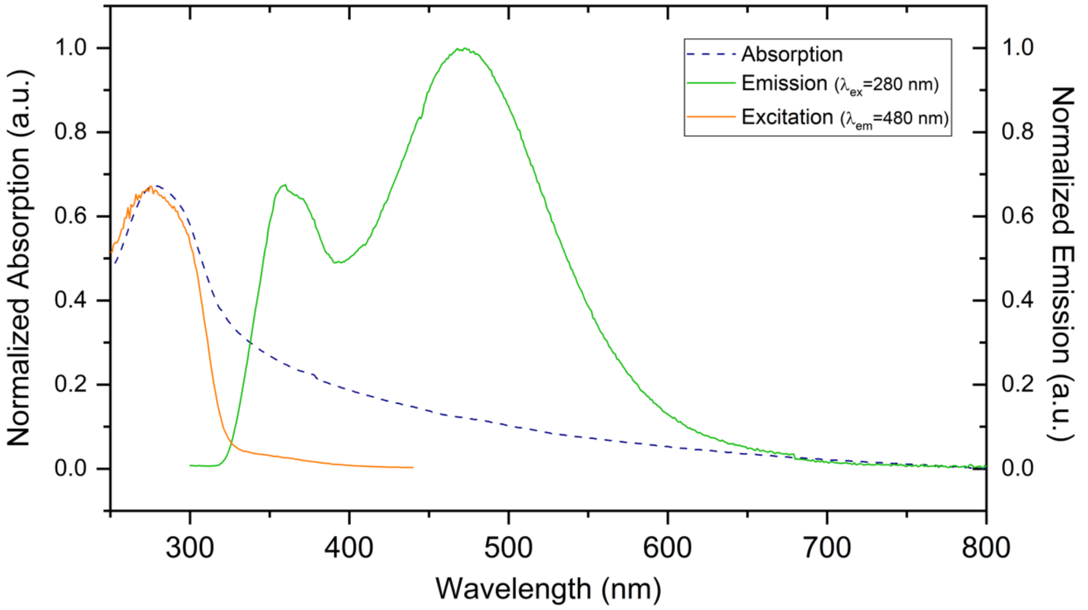

Figure S37. Absorption, excitation, and emission spectra of the empty **TPPM** phase in the solid state.

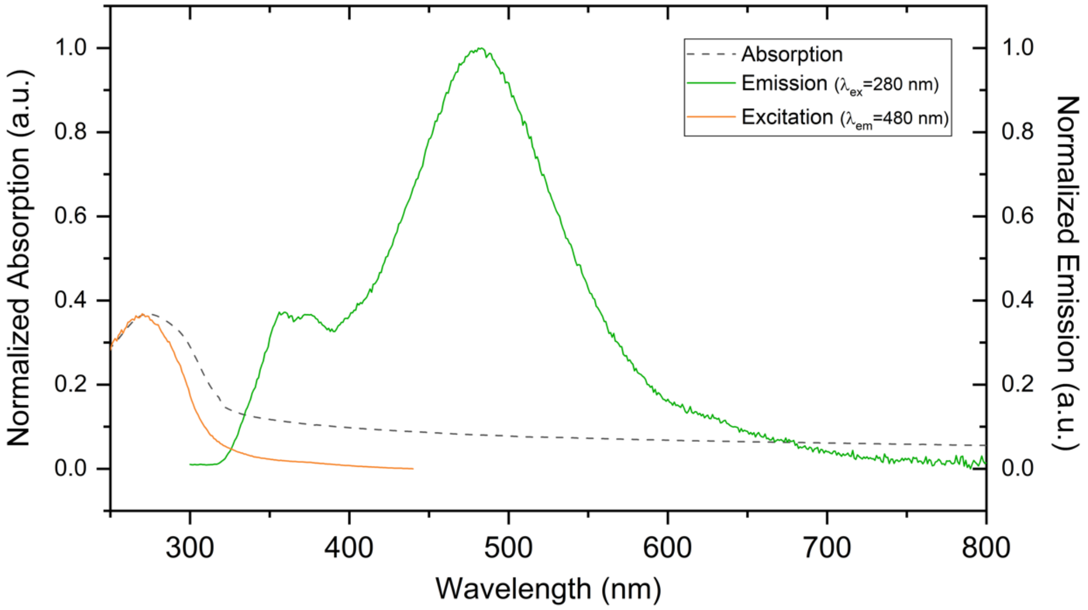

Figure S38. Absorption, excitation, and emission spectra of **TPPM·$CHCl_3$** in the solid state.

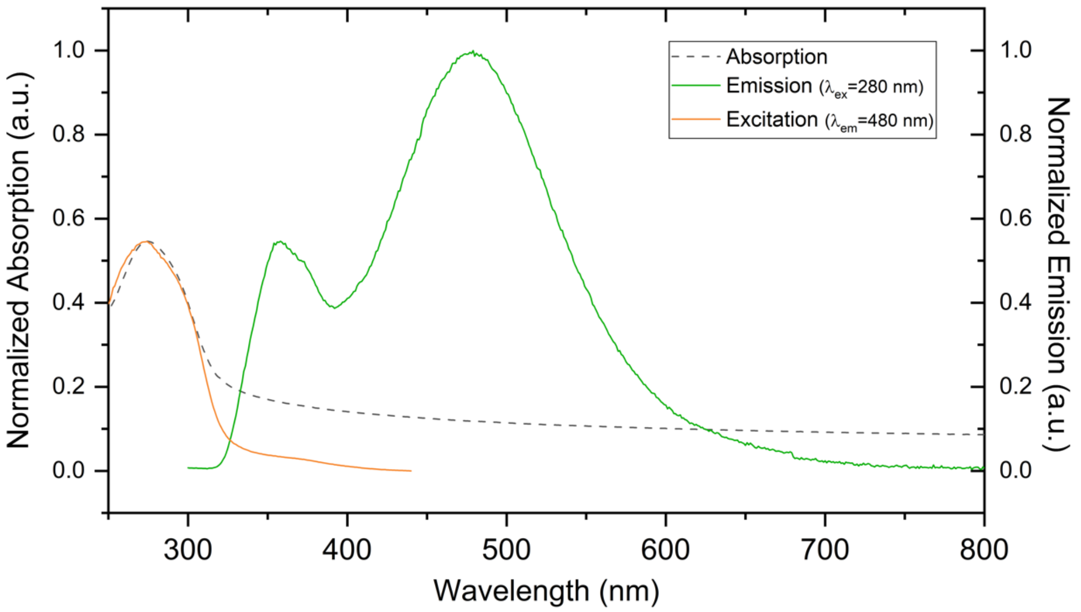

Figure S39. Absorption, excitation, and emission spectra of the **TPPM·C_6H_6** phase in the solid state.

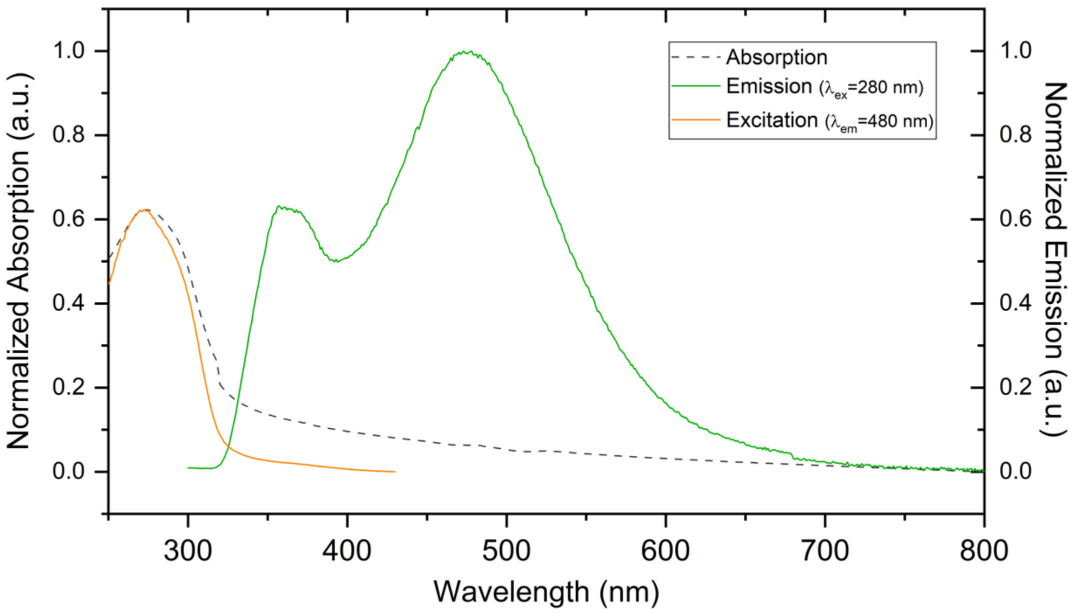

Figure S40. Absorption, excitation, and emission spectra of **TPPM·$PhCH_3$** in the solid state.

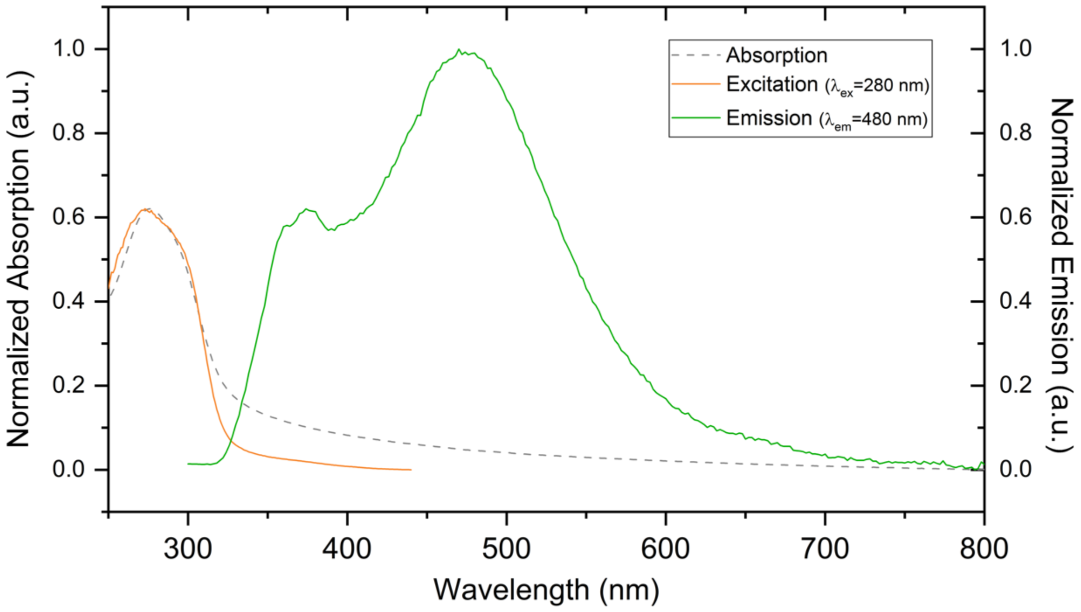

Figure S41. Absorption, excitation, and emission spectra of **TPPM·C_2HCl_3** in the solid state.

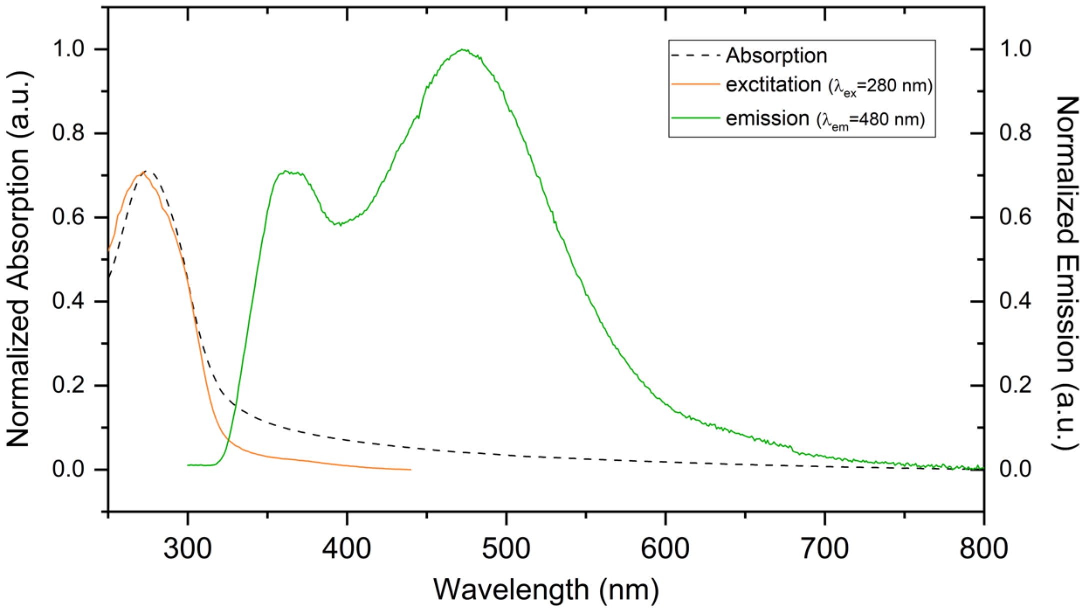

Figure S42. Absorption, excitation, and emission spectra of **TPPM·C_6H_{12}** in the solid state.

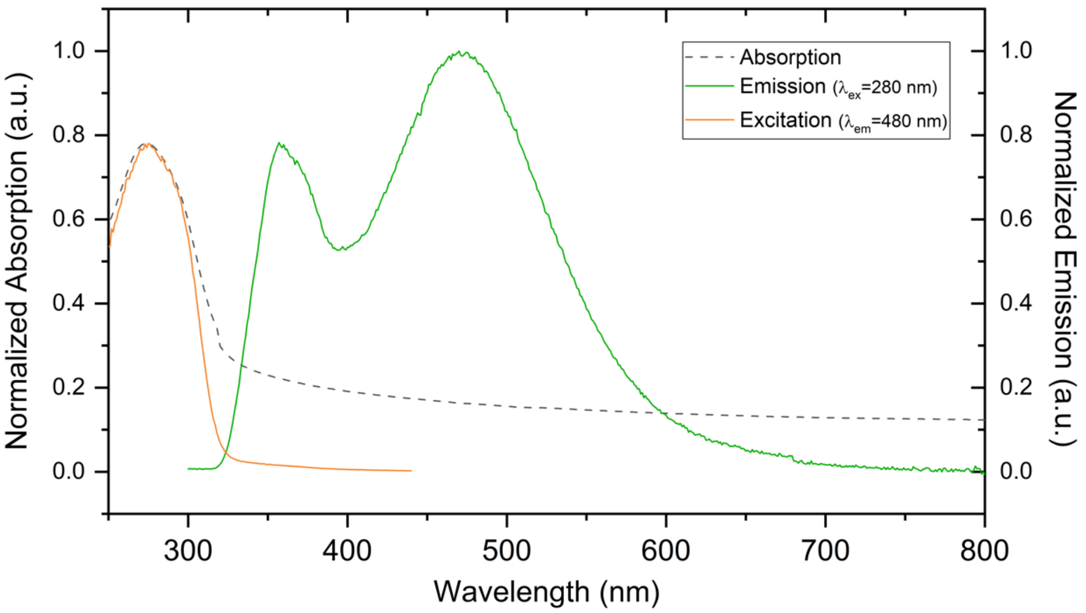

Figure S43. Absorption, excitation, and emission spectra of **TPPM·DCE** in the solid state.

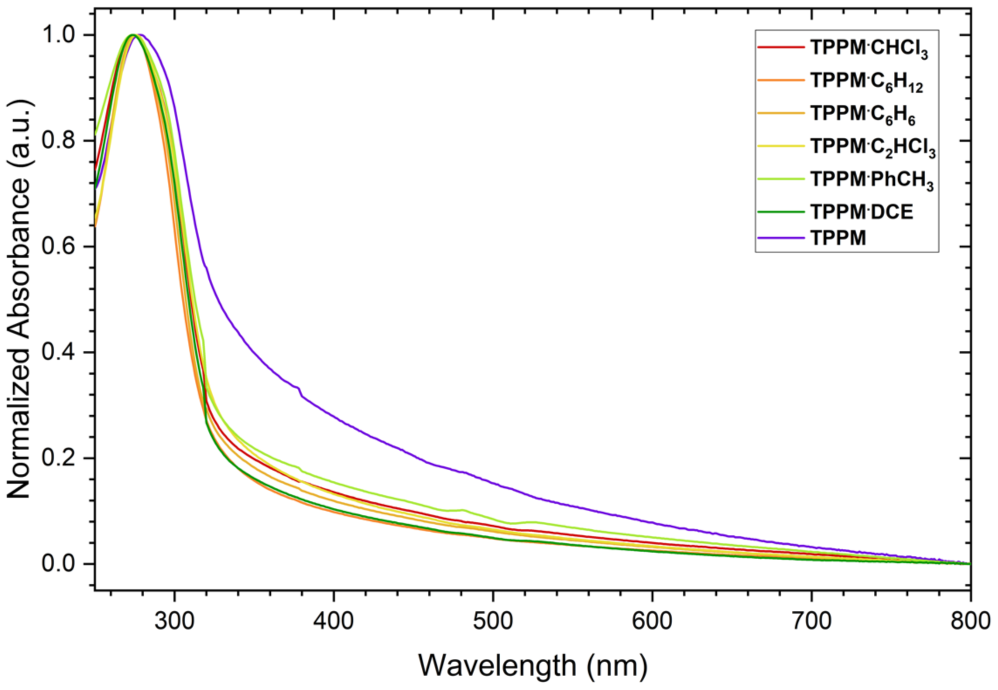

Figure S44. Comparison of the absorption spectra at the solid state for the different **TPPM** phases.

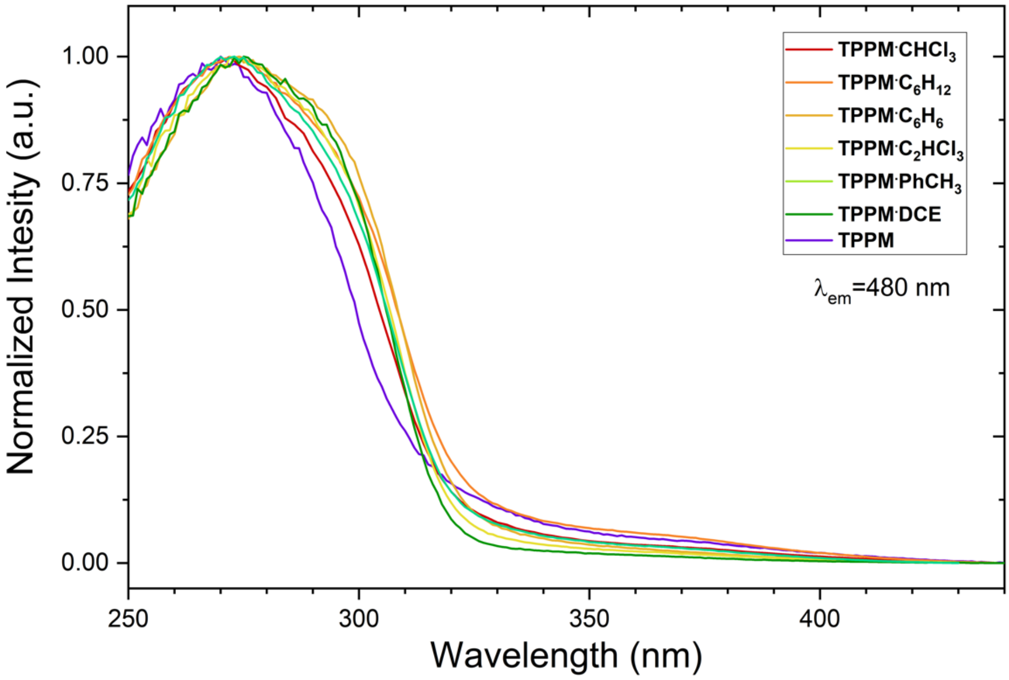

Figure S45. Comparison of the excitation spectra at the solid state for the different **TPPM** phases.

Table S5. Emission Ratio value for each **TPPM** phase characterized by solid-state spectroscopic methods. To perform a statistical analysis, the emission spectra were collected for three different samples.

TPPM Crystal Phase	Ratio I_{480nm}/I_{360nm}	Mean Value	Standard Deviation
**TPPM·$CHCl_3$**	2.21035	2.71874	0.49312
	2.75085		
	3.19502		
**TPPM·C_6H_{12}**	1.56512	1.79224	0.5353
	1.40794		
	2.40366		
**TPPM·C_6H_6**	1.49289	1.79589	0.26564
	1.90603		
	1.98874		
**TPPM·C_2HCl_3**	1.6129	1.4968	0.12073
	1.50557		
	1.37193		
**TPPM·$PhCH_3$**	1.5791	1.70348	0.17374
	1.94918		
	1.58215		
**TPPM·DCE**	1.64479	1.72997	0.20764
	1.96666		
	1.57846		
**TPPM**	1.17315	1.31384	0.15554
	1.48086		
	1.2875		

Table S6. Significance levels for each group obtained by one-way ANOVA calculation by Tukey Test, using 0.05 as significance level.

	**TPPM·$CHCl_3$**	**TPPM·C_6H_{12}**	**TPPM·C_6H_6**	**TPPM·C_2HCl_3**	**TPPM·$PhCH_3$**	**TPPM·DCE**	**TPPM**
**TPPM·$CHCl_3$**							
**TPPM·C_6H_{12}**	0.04142						
**TPPM·C_6H_6**	0.04246	1					
**TPPM·C_2HCl_3**	0.0054	0.91073	0.90609				
**TPPM·$PhCH_3$**	0.02251	0.99983	0.99979	0.98278			
**TPPM·DCE**	0.02703	0.99998	0.99997	0.96899	1		
**TPPM**	0.00157	0.55813	0.5501	0.99074	0.75143	0.69557	

8. References

1. Kitagawa, H.; Ohtsu, H.; Kawano, M. Kinetic Assembly of a Thermally Stable Porous Coordination Network Based on Labile CuI Units and the Visualization of I_2 Sorption. *Angew. Chem. Int. Ed.* **2013**, *52*, 12395-12399.
2. (a) SADABS Bruker AXS; Madison, Wisconsin, USA, 2004; SAINT, Software Users Guide, Version 6.0; Bruker Analytical X-ray Systems, Madison, WI, **1999**; (b) Sheldrick, G. M. SADABS v2.03: Area-Detector Absorption Correction. University of Göttingen, Germany, **1999**.
3. Sheldrick, G. M. SHELXT - Integrated space-group and crystal-structure determination. *Acta Cryst.* **2015**, *A71*, 3-8.
4. Sheldrick, G. M. A short history of SHELX. Acta Cryst. **2008**, A64, 112-122.
5. Farrugia, L. J. WinGX Suite for Small-Molecule Single-Crystal Crystallography. *J. Appl. Crystallogr*. **1999**, *32*, 837-838.
6. Caputo, C. B.; Vukotic, V. N.; Sirizzotti, N. M; Loeb, S. J. A tetrapyridine ligand with a rigid tetrahedral core forms metal–organic frameworks with PtS type architecture. *Chem. Commun*. **2011**, *47*, 8545-8547.
7. Gaussian 09, Revision D.01, Frisch, M. J.; Trucks, G. W.; Schlegel, H. B.; Scuseria, G. E.; Robb, M. A.; Cheeseman, J. R.; Scalmani, G.; Barone, V.; Petersson, G. A.; Nakatsuji, H.; Li, X.; Caricato, M.; Marenich, M.; Bloino, J.; Janesko, B. G.; Gomperts, R.; Mennucci, B.; Hratchian, H. P.; Ortiz, J. V.; Izmaylov, A. F.; Sonnenberg, J. L.; Williams-Young, D.; Ding, F.; Lipparini, F.; Egidi, F.; Goings, J.; Peng, B.; Petrone, A.; Henderson, T.; Ranasinghe, D.; Zakrzewski, V. G.; Gao, J.; Rega, N.; Zheng, G.; Liang, W.; Hada, M.; Ehara, M.; Toyota, K.; Fukuda, R.; Hasegawa, J.; Ishida, M.; Nakajima, T.; Honda, Y.; Kitao, O.; Nakai, H.; Vreven, T.; Throssell, K.; Montgomery, J. A.; Peralta, Jr., J. E.; Ogliaro, F.; Bearpark, M.; Heyd, J. J.; Brothers, E.; Kudin, K. N.; Staroverov, V. N.; Keith, T.;. Kobayashi, R.; Normand, J.; Raghavachari, K.; Rendell, A.; Burant, J. C.; Iyengar, S. S.; Tomasi, J.; Cossi, M.; Millam, J. M.; Klene, M.; Adamo, C.; Cammi, R.; Ochterski, J. W.; Martin, R. L.; Morokuma, K.; Farkas, O.; Foresman, J. B., Fox, D. J. Gaussian, Inc., Wallingford CT, 2016.

11. Petříček, V.; Dušek, M.; Palatinus, L. *Z.* Crystallographic Computing System JANA2006: General features. *Kristallogr*. **2014**, *229*, 345-352.

12. Maglic, J. B.; Lavendomme, R. MoloVol: an Easy-to-Use Program for Analyzing Cavities, Volumes and Surface Areas of Chemical Structures: *J. Appl. Cryst*. **2022**, *55*.

13. Macrae, C. F.; Sovago, I.; Cottrell, S. J.; Galek, P. T. A.; McCabe, P.; Pidcock, E.; Platings, M.; Shields, G. P.; Stevens, J. S.; Towler, M.; Wood. P. A. Mercury 4.0: from visualization to analysis, design and prediction. *J. Appl. Cryst*., **2020**, *53*, 226-235.
